# Cultural adaptation and content validation of the WHO BeSD framework for HPV vaccination in Pakistan: A two-phase Delphi and cognitive interview study

**DOI:** 10.1371/journal.pone.0335358

**Published:** 2026-02-17

**Authors:** Khola Noreen, Samina Naeem Khalid, Mehreen Noor, Saba Maryam, Shahzad Ali Khan, Abdul Momin Rizwan Ahmad

**Affiliations:** 1 Department of Community Medicine & Public Health, Rawalpindi Medical University, Rawalpindi, Pakistan; 2 Public Health, Health Services Academy, Islamabad, Pakistan; 3 Department of Human Nutrition and Dietetics, NUST School of Health Sciences, National University of Sciences & Technology (NUST), Islamabad, Pakistan; 4 Department of Health Sciences, University of York, York, United Kingdom; Federal University Otuoke, NIGERIA

## Abstract

**Background:**

The BeSD Framework (Behavioral and Social Drivers of Vaccination), developed by the World Health Organization, provides a structured approach to understanding the behavior and social drivers of vaccine uptake. However, it’s direct use in diverse cultural backgrounds especially in the context of HPV vaccination, is not yet established. Therefore, this study aimed to culturally adapt and establish content validity of the BeSD framework for assessing behavioral and social drivers of Human papillomavirus (HPV) vaccination in Pakistan.

**Methods:**

A two-phase multi-method cross-cultural adaptation with expert content validation and cognitive interviews was used following the emic–etic paradigm and globally accepted Beaton’s cross-cultural adaptation guidelines. The initial items of the BeSD-HPV tool were translated into Urdu through forward–backward translation and expert synthesis. Content validity was assessed using a two-round Delphi method with ten multidisciplinary experts, who rated items on a four-point relevance and clarity scale. Consensus was a priori defined as item-level content validity index scores of ≥0.80. A scale-level content validity index score was also calculated. Response-process validity was then evaluated through cognitive interviews with 30 caregivers of adolescent girls using think-aloud and probing techniques to assess comprehension and cultural appropriateness.

**Results:**

Ten experts participated in both rounds of the online Delphi study. At the completion of the study, out of the initial 75 items, two items were dropped in round one and three items were dropped in round 2. Out of the total 73 left in round 1, 28 items were accepted by all experts without revision, 42 were revised in rounds 2 and 3 were dropped in round 2, resulting in a 70-item BeSD-HPV tool. The scale-level content validity index score for the final 70-item instrument was 0.96. Cognitive interviews using the think-aloud technique and probing were conducted with parents/caregivers of girls aged 9–16 years. A total of 30 interviews were conducted. Seventeen items categorized as having minor or major issues were revised by the authors based on participant feedback to improve comprehension.

**Conclusion:**

The adapted BeSD-HPV tool demonstrated strong content and response-process validity for the Pakistani context. This culturally sensitive instrument will support future large-scale studies to determine the construct validity and reliability of the BeSD-HPV, thereby providing a validated tool to assess drivers of HPV vaccine acceptance and inform future context-specific demand generation strategies.

## Introduction

Cultural adaptation is the “systematic process of modifying a tool to ensure both linguistic equivalence and cultural relevance, so that the content is meaningful, appropriate, and valid for use in a different cultural setting” [1]. Cultural adaptation of health-related measurement tools is critical to safeguarding that constructs developed in one cultural landscape retain their credibility, applicability, clarity, and transparency when applied in another. Nowadays, a growing corpus of literature from several academic fields explains how to accomplish successful cross-cultural adaptation by going through several validation stages [2,3]. The most popular procedures are back-translation, translation, and content validation with input from content experts [4].

The BeSD Framework was originally developed by the World Health Organization (WHO) to capture the behavioral and social drivers of immunization across various global settings [5]. However, when applied to local contexts, the BeSD Framework may underrepresent several factors embedded in multifaceted cultural, social, and linguistic structures. The literature on cross-cultural adaptation emphasizes that contextualizing each item to conform to local cultural meanings, social norms, and lived experiences is necessary to achieve semantic, idiomatic, experiential, and conceptual equivalence; merely translating language one-to-one does not serve the purpose [1,6].

In Pakistan, vaccination decisions are strongly shaped by patriarchal influence, direction of religious representatives, gender-based norms, influential community figures, and myths and taboos prevailing in society [7]. Constructs such as “trust in healthcare providers,” “social influence,” or “motivation to vaccinate” may not hold equivalent meaning in the sociocultural context of Pakistan having diverse cultural frameworks. The original BeSD framework might not accurately represent complex sociocultural realities, context-specific health system dynamics, or regionally significant drivers of vaccination uptake. This emphasizes the need for the WHO’s Behavioral and Social Drivers (BeSD) framework, primarily designed to evaluate drivers of vaccination behavior, to undergo meticulous cultural adaptation before being applied across varied sociocultural settings such as Pakistan. Without this process, tools may overlook culturally embedded meanings, practices, and decision pathways, leading to measurement and misclassification errors, validity and reliability issues, and limited generalizability [1,8].

The emic–etic paradigm provides a comprehensive theoretical framework for such adaptation [9]. The emic perspective emphasizes indigenous viewpoints, emphasizing culturally grounded understandings, while the etic perspective emphasizes universal constructs that support comparability across cultural context [10]. In tool adaptation, the original instrument reflects an imposed etic, serving as the foundational framework (original BeSD). Emic exploration, through qualitative research, stakeholder consultation, and participatory engagement helps to identify culturally relevant constructs that may be missing or inadequately represented. Integration of these perspectives produces a derived etic instrument (BeSD-HPV), which preserves cross-cultural comparability while incorporating context-specific drivers of behavior. For HPV vaccination in Pakistan, such locally relevant drivers may include fertility implications, religious endorsement, and spousal or parental influence in health choices.

Established guidelines proposed by Beaton et al. (2000) and WHO (2016) reinforce this universal approach. Beaton et al. (2000) describe a six-step process for the cross-cultural adaptation of self-report measurement tools, including forward translation, reconciliation, reverse translation, expert panel evaluation, and pilot testing (cognitive interviews), and documentation thereby, maintaining linguistic precision and conceptual alignment [1]. Similarly, WHO (2016) emphasizes iterative testing, expert review, and field validation to balance contextual appropriateness with cross-cultural comparability.[11] By embedding these processes within an emic–etic framework, adaptation moves beyond maintaining linguistic integrity towards cultural resonance and strong psychometric validity,

In the present study, the BeSD framework for HPV vaccination was adapted for use in Pakistan through this dual approach: (1) Methodological robustness informed by Beaton et al.’s and WHO’s adaptation guidelines, and (2) theoretical grounding using the emic–etic paradigm. This ensured that, while the adapted instrument preserved its global comparability, it also incorporated new items derived from emic insights, capturing the unique sociocultural realities that influence vaccine uptake in Pakistan. Such integration not only enhances the validity of the adapted instrument but also enhances its applicability in informing culturally responsive public health strategies and policies.

### Rationale

The study employed a two-phase multi-method cross-cultural adaptation with expert content validation grounded in an emic-etic framework, based on internationally recognized and globally accepted guidelines proposed by Beaton et al. and the WHO, to maintain the etic integrity of the BeSD framework while incorporating emic characteristics important in Pakistan [1,11].

Guidelines proposed by Beaton et al. were used to maintain semantic, conceptual, and experiential equivalence. Bilingual translators used forward and backward translations to ensure semantic equivalency, resolving discrepancies by consensus to maintain in-depth meaning rather than exact literal wording of the language. Conceptual equivalency was maintained by mapping each item into the existing four BeSD domains and evaluating its relevancy and clarity through an iterative process involving Delphi consensus rounds. The rationale of using delphi technique was to culturally adapt BeSD framework, instead of using original tool directly was because of complexity involved in vaccine related decision making in sociocultural context of Pakistan. Cultural phenomenon influencing the vaccination uptake include myths, taboos, patriarchal hierarchies, limited female autonomy, sociocultural and linguistic disparities influencing vaccine behavior, which warrants expert validation through iterative Delphi rounds to ensure conceptual equivalency.

Experiential equivalency was maintained through incorporating findings of formative qualitative research and cognitive interviews. Finally, the tool was refined through an iterative process of expert review and pilot testing, thus ensuring cross-cultural comparability with BeSD applications in other contexts while remaining culturally valid in Pakistan. The entire process involved rigorous, methodical cultural adaptation, translation and content validation of the WHO Behavioral and Social Drivers (BeSD) framework for application in the Pakistani context, while maintaining cross-country comparability and incorporating culturally relevant behavioral social determinants.

### Objectives

- To culturally adapt the WHO BeSD framework for HPV vaccination and establish content and response-process validity for the Pakistani context and produce a final BeSD-HPV instrument.

## Materials & methods

### Theoretical base for cultural adaptation

The WHO BeSD tool offers a strong global framework, but its constructs, terminologies, and response categories need to be carefully evaluated to ensure the inclusion of cultural resonance within diverse sociocultural context of Pakistan. Direct adaptation imposes the risk of ignoring complex local factors such as different trust dynamics, gendered decision-making, linguistic disparities, cultural and context -specific misconceptions.

The cultural adaptation of the BeSD tool was guided by an integrated emic-etic framework, operationalized through various iterative steps. First, an imposed etic perspective was applied by using the original BeSD framework and its four universal domains namely thinking and feeling, social processes, motivation, and practical issues, which were used as the conceptual anchor for translation and preliminary item mapping. Second, the inclusion of cultural resonance was ensured through emic exploration using formative qualitative research, sociolinguistic adaptations, and expert validation via Delphi consensus. Delphi method was used to ensure that experts reach an agreement on necessary changes while maintaining conceptual clarity, this method allowed systematic, iterative engagement with Pakistani experts in the fields of Public Health, Behavioral Science, Anthropology, and Immunization programs to give their valuable insights and agreed upon unanimous decision in adaptation of existing framework in Pakistani context. Finally, a derived etic perspective was achieved through cognitive interviews and expert feedback, which were used to assess content relevance and clarity and determine the addition, modification, or deletion of items within the four BeSD domains. This process resulted in a modified BeSD-HPV instrument that preserved global comparability while incorporating cultural validity for the Pakistani setting. This methodical procedure ensured that the instrument is both socially and scientifically grounded by striking a balance between contextual meaning and reliability.

### The research instrument (from permission to translation)

Preliminary version of BeSD HPV tool developed after contextualizing original BeSD framework through rigorous qualitative research and literature review (submitted for publication and under review at PLOS ONE ref: PONE-D-25–55196) was subjected to cultural adaptation and content validation in the current study. The original BeSD framework, which contains a quantitative survey-based questionnaire and qualitative interview guides, was used to develop a preliminary version of BeSD-HPV after taking permission from relevant stakeholders. The research team sent a formal request to Lisa Menning at WHO Headquarters via email seeking permission to use the BeSD framework, along with qualitative interview guides for cultural adaptation.

### Rationale of Delphi technique

The research team used Delphi technique to culturally adapt BeSD framework, instead of using original tool directly owning to complexity involved in vaccine related decision making in sociocultural context of Pakistan. Cultural phenomenon influencing the vaccination uptake include myths, taboos, patriarchal hierarchies, limited female autonomy, sociocultural and linguistic disparities influencing vaccine behavior, which warrants cultural adaptation of behavioral and Social Drivers (BeSD) framework of Vaccination in Pakistani context.

The study employed the Delphi process, which entails formative qualitative research that guided item generation through a methodological and rigorous manner assuring rigor and contextual relevance. The qualitative research involved the lived experience and voices of potential stakeholders aligning with circumstantial relevance of HPV vaccination in context of Pakistan. Braun and Clarke’s six steps thematic analysis was used with deductive coding to align themes and subthemes with existing domains and inductive coding to align newly emerging themes with new domain labelled as “Cultural Integration”. Themes and subthemes were mapped to new constructs under existing four BeSD domain in addition to new domain “cultural integration”, theme led the generation of new items in alignment with specific quotes charted with its justification (attached as S1 File).

### Data collection procedure

Data was collected after taking informed consent. Researchers asked participants for informed verbal consent after explaining study objectives, detailed methodology, study procedure, voluntary participation and participant’s right to withdraw without any consequences. All potential participants were also given detailed elaboration of potential risks and benefits in lay language using simple and easy understandable Urdu. Participants were also informed in detail that their participation is completely voluntary, and they can choose to withdraw at any point without repercussions. Once participants showed willingness to participate and consented to get enrolled in study, copy of informed consent was read to them. Participants were given an opportunity to ask questions, clarify queries and express their concerns if any. Following this, those who give final authorization were given informed written consent to get it signed and keep its hard copy for their record.

Participants were informed that data is being collected from them for research purpose and it will be used for publication purposes. Participants were fully assured of anonymity and confidentiality of data and guaranteed that their personal information will not be disclosed at any point during study. Additionally, data collected during in-depth interviews and focus group discussion was deidentified by assigning a specific code to everyone thus assuring anonymity and confidentiality. Other potential identifiers like age, workplace name, gender, sample size, postal address, email address, specific dates were deidentified to ensure complete anonymity and confidentiality.

### Ethical approval

The qualitative data that guide item development was collected from February 2025 to August 2025 at District Rawalpindi and Islamabad Capital Territory from Northern Punjab and District Bahawalpur from Southern Punjab. Ethical Approval was obtained from the Ethical Review Board (ERB) of Health Services Academy. Approval was granted on February 24, 2025, under IRB approval number (00015/HAS/PhD 2022) from Health Services Academy. Ethical approval was also obtained from Ethical Review Board of Rawalpindi Medical University through Letter No Ref. No. 843/IREF/RMU/2024 dated August 13, 2024. The study was conducted after taking informed written consent and in accordance with the policies and procedures established by the Graduate Research Management Council (GRMC) of Health Services Academy HSA Research and Ethical Committee

### Evidence synthesis and item mapping

#### Evidence synthesis.

Findings from qualitative research, including focus group discussions (FGDs) and in-depth interviews (submitted for publication and under review at PLOS ONE ref: PONE-D-25–55196), guide the item generation, mapped to the existing four domains. Emergent themes guided the incorporation of a new construct (cultural integration) in addition to the existing four domains.

#### Generation of item bank.

We compiled an item bank comprising: 1. original BeSD items(retain) 2. contextually misfitting (need revision) and 3. new culturally relevant items (addition). Item mapping was conducted with justification and rationale for mapping each item to a specific domain. (manuscript submitted for publication and under review at PLOS ONE ref: PONE-D-25–55196). Once a preliminary version of HPV specific BeSD tool (BeSD-HPV) was subjected to a cultural adaptation process as per guidelines by Beaton et al. and WHO to maintain the etic integrity of the BeSD framework while including emic characteristics that are important in Pakistan [1,11].

### Phase 1: Application of Beaton’s six-step framework for BeSD-HPV adaptation

**Fig 1:**
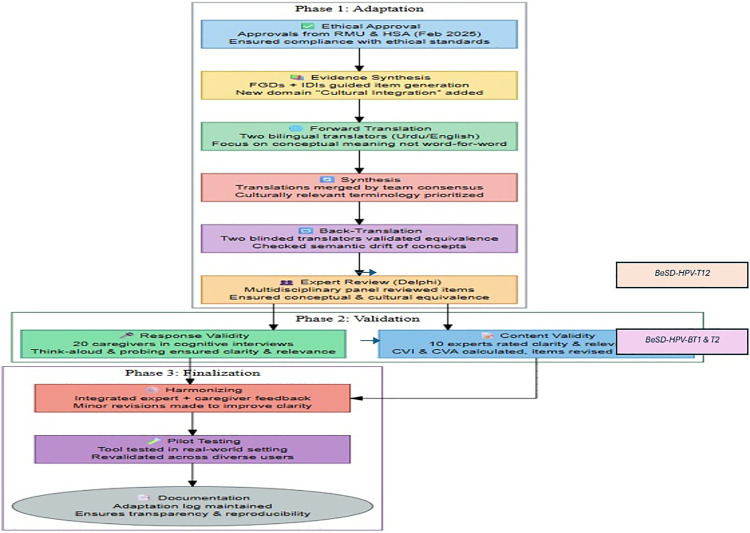
Beaton’s six-step framework for cultural adaptation of BeSD-HPV.

Fig 1 presents the flow chart for the development and validation process of the adapted BeSD -HPV.

#### Step 1: Translating the BeSD-HPV Into Urdu (forward translation).

To ensure linguistic accuracy, two independent translators (HA and MK), native Urdu speakers with academic proficiency in English Language and knowledgeable about Public Health jargon and terminologies, translated the BeSD-HPV item into Urdu (the National language of Pakistan). Their adaptations aim to offer equivalence from a Public Health perspective, and they may result in a translation that offers a more trustworthy equivalence from a measurement standpoint. Instead of translating word for word, the emphasis was on communicating conceptual meaning (for example, “confidence in vaccination” was translated as “yakeen” to represent faith in the medical system and doctors). Once a translation was confirmed, BeSD-HPV, now termed as BeSD-HPV-T1 & BeSD-HPV-T2.

#### Step 2: Synthesis of Translations.

The research team compared the two forward translations, discussed inconsistencies, and synthesized them into a single version. The research team (SN, KN & SAK) ensured that terms were linguistically relevant, culturally appropriate, and understandable in the Pakistani context. The new synthesized Urdu version is labeled as (BeSD-HPV-T12).

#### Step 3: Translating the BeSD-HPV-U Into English (backward translation).

Likewise, two independent translators (SR& MI), who were totally blind to the original version (BeSD-HPV), performed reversed translation, resulting in translation back into the original language (BeSD-HPV-BT1 & BT2). Among two translators, one was a psychologist and other was a professor of English language; both were proficient in English language and purposely selected from non-medical backgrounds. This is to elicit unexpected meanings of the items in the translated questionnaire and to avoid information bias, thus increasing the likelihood of highlighting the imperfections. This is a validity check procedure to ensure that the translated version accurately reflects the content of the original versions. This step also highlights conceptual errors, unclear wordings, and gross inconsistencies in the translation and ensures consistency in translation process [12].

This process guaranteed that the items had retained their conceptual integrity and indicated phrases that had undergone semantic drift (for example, “autonomy” changing its meaning from “individual choice” to “khud mukhtari”). Once the research team received both translated versions, they synthesized them into a single English version, and an expert panel Review (Delphi) was conducted to validate it.

#### Step 4: Expert committee review tool validation via Delphi technique:.

Step 4 comprises 2 stages

1. Establishing content validity
2. Response process validity

***Stage 1: Establishing content validity***

**Step 1: Preparing Content Validation Form**

The first step of content validation is to prepare a content validation form to ensure that experts have a clear understanding and clear expectations of the task. A content validity form was prepared using JotForm, which contained detailed information about the purpose of the study, along with details of informed consent and volunteer participation. The definition of each domain was given for clear understanding. The participants were given detailed information about purpose of study and instructions were provided to access the scale on relevance and clarity along with rating scales.

**Step 2: Formation of a multidisciplinary panel**

A multidisciplinary expert panel relevant to subjects under study was requested for content validation of the BeSD-HPV Tool. A committee with diverse expertise in various fields was constituted to ensure conceptual, semantic, experiential, and idiomatic equivalence.

The study was conducted in two parts. The first part includes the Delphi round, which includes 10 experts in the fields of research, public health, anthropology, medical education, gynecology & obstetrics, and pediatricians. Details of experts and their qualifications are given in S2 File. The link to JotForm was shared with experts through an invitation email.

**Step 3: Establishing content validity**

Content validity can be conducted through both face-to-face and non-face-to-face approaches. The most important factors required to be considered are time, cost, and response rate. Since the expert panel in our study belongs to a diverse range of professional backgrounds, both at the national level and international level, with varying levels of professional commitment and variation in time zones, it was difficult to get all experts together. Therefore, time, cost, logistics and response rate were challenging factors in organizing a face-to-face approach; consequently, a non-face-to-face approach was chosen to achieve the highest response rate. Literature also supports the notion that the non-face-to-face technique is highly efficient when accompanied by a systematic follow-up to increase the response rate and time.

#### Sample size justification & sampling techniques.

Delphi technique was used in this study to assess clarity, relevance, establish content validity and cultural appropriateness of adapted BeSD framework and to reach expert consensus.

The sample size for Delphi study is determined by methodological guidelines for consensus studies. Focus is on expertise and diversity rather than achieving statistical power criteria. Literature supports with evidence that Delphi panel usually comprises of 2–10 experts which are adequate to achieve consensus while ensuring adequate iteration cycle [13,14]. This also helps to minimize response burden while maintaining alignment with international standards of Delphi for content validation. Considering the complex and multidimensional nature of HPV vaccine uptake, diverse sample size of ten experts comprising of Public Health, Medical Educationist, Pediatrician, Gynecologist, behavior scientist, Anthropologist, program managers of vaccination were recruited from June 2025 to July 2025 after obtaining informed consent. Purposive sampling was employed to ensure the inclusion of people with the necessary expertise to offer expert input on the relevance and clarity of the items. Through professional networks, the experts were approached personally and elaborated on the study in detail through information shared via an invitation email. The recruitment of ten experts aligns with accepted standards for content validity studies, which states that a panel of five to ten subject matter experts is sufficient to assess clarity and relevance of the tool items. It is suggested that a minimum of two experts is required for content validation; however, most of references recommend a minimum of six experts. Evidence from the literature suggests that the minimum number of experts required for content validation should be at least six and not exceed 10 [13–16]. Considering this recommendation, we selected 10 experts for content validation in our study.

#### Inclusion criteria.

- Public Health professionals with a minimum of 5 years of experience
- Medical Educationist with a good command of psychometric
- Having an administrative or leadership role in research, gynae & obs, pediatrics
- Anthropologist with expertise in behavior/social sciences

#### Data collection method.

Two Delphi rounds were planned prior to the study, taking into consideration the busy schedules of experts, this is also consistent with published evidence that two to four rounds are sufficient to achieve consensus in Delphi studies. All 10 experts (100%) responded in rounds 1 and 2, thereby ensuring no attrition bias. Participants were given one week to complete both rounds 1 and 2 and submit their responses through JotForm (https://form.jotform.com/252220848734054).

JOT Form with Expert Feedback zip folder has been uploaded as S3 File.

**A. Delphi Round 1**

***Distribution of Online Proforma:***

The link for the content validation proforma was shared with experts through JotForm. After clicking the survey link, participants were directed to an online platform. Before filling out the proforma, participants were asked to confirm their consent to participate. Participants were given a 5-day time frame for completing round 1. All participants shared results within the prescribed time frame.

***Procedures***

***Rating Scales and Scoring Approach***

The experts rated each item for relevance and clarity on 4-point Likert scale as follows:

**Relevance** – whether the item reflects the intended construct in the local context

- **Relevance Scale:** Relevance was assessed on 4-point Likert scale 1= Not relevant,2 = Somewhat relevant, 3 = Quite relevant, 4 = Very relevant
- **Clarity**—whether the wording is clear, concise, and culturally appropriate
- **Clarity Scale:** Clarity was assessed on 4-point Likert scale 1= Not clear, 2 = Somewhat clear, 3 = Quite clear, 4 = Very clear

***Binary Coding & I-CVI Calculation***

For CVI calculations, scores were dichotomized:

- Ratings of 1 or 2 = 0 (non-agreement)
- Ratings of 3 or 4 = 1 (agreement)

This binary score allows for the quantification of expert consensus and in accordance with established CVI guidelines.

***Item-Level Agreement (I-CVI)***

The Item-Level Content Validity Index (I-CVI) was calculated using following formula:

- I-CVI = (Number of experts in agreement)/ (Total number of experts)

Interpretation thresholds:

-  ≥ 0.80 on both relevance and clarity → item retained
-  ≥ 0.80 with qualitative suggestions → item flagged for revision
-  < 0.80 → item deleted

***Scale-Level Agreement (S-CVI)***

The Scale-level Content Validity Index (S-CVI) was calculated using following formula:

- CVI=(Sum of I-CVI scores)/(Total number of items).

S-CVI reflects the overall content validity of the instrument.

- S-CVI/Ave ≥ 0.90: This indicates excellent content validity of instrument

***Qualitative feedback***

An open-text field accompanied each item where experts could give qualitative feedback including:

- Revision, rewording
- Changes in domain names or definitions
- Identification of culturally inappropriate content
- Suggestions for adding or removing items
- Insights from field experience with HPV vaccination in Pakistan (if applicable)

**B. In between two rounds:**

Feedback received from experts during round 1 was compiled and incorporated during this phase.

***Compilation of quantitative and qualitative feedback:***

The feedback of experts during round 1 was the compiled and incorporated during this phase, feedback includes following:

- item-level content validity index (I-CVI)
- mean rating for each item
- anonymized aggregated qualitative comments
- conflicting remarks and statements

***Sharing anonymized feedback to experts***

After incorporation of compiled feedback, anonymized feedback was shared to each expert

***Individualized consultation with experts (where needed)***

One to one consultation was done through email, virtual meetings through zoom and whatsapp calls to clarify socio culturally sensitive issues.

***Item Revision:***

On basis of feedback items were revised to ensure

- Cultural relevance with Pakistani context
- Enhance conceptual clarity
- Removal of duplication
- Enriched alignment with BeSD construct

***C. Round 2:***

After all feedback incorporated and revisions completed, instrument was shared again for round 2 ratings. All revised items were re-rated by expert panel for relevance and clarity using same structured rating scale. This phase helped in further refinement, improvement, resolution of overlapping items and further Item Reduction

## Results

### Round 1 findings

Seventy-five were evaluated for content validation in round 1.

- Two items were removed: I-CVI < 0.80 across both dimension (relevance & clarity)

Out of remaining 73 items:

- 28 items met acceptance threshold without modification: I-CVI ≥ 0.80 across both dimensions
- Interpretation: reflect strong contextual relevance and conceptual clarity
- Remaining 45 items met quantitative threshold but generated qualitative suggestion by experts regarding phrasing, expression and cultural application.
- Interpretation: These items were conceptually relevant but required sociolinguistic adaptation ensuring inclusion of cultural resonance in alignment with emic exploration

The S-CVI for Round 1 was 0.88, indicating good but improvable content validity.

#### Interpretation.

High I-CVI scores of items represent conceptual agreement with original BeSD construct in alignment with etic concept, yet expert opinion highlights that significant number of items need revision thus underscoring the importance of cultural adaptation of BeSD framework by incorporating emic characteristics relevant to sociocultural context of Pakistan

### Integration of Quantitative and Qualitative feedback in between two rounds

Substantive disagreements on item wording or relevance were discussed among the research team, and a proposed revision was crafted. This proposed revision, along with the rationale, was then shared with the relevant experts for final confirmation before inclusion in Round 2. Experts received anonymized aggregated feedback in between rounds. This iterative feedback enabled specialists to reevaluate their conclusions, considering the group trend without compromising anonymity.

#### Interpretation.

The merger of quantitative scores with qualitative explanation promote consensus by reducing divergent interpretations in alignment with Delphi theory. Experts reconsider their initial judgement and reconcile findings based on group trend which allowed incorporation of emic perspective while maintaining etic paradigm of original BeSD framework.

### Delphi Round 2

#### Deletion of Items.

A total of three items deleted during round 2:

- Two items having I-CVI > 0.80 were deleted (item 35 overlapped with item 62 and item 50 overlapped with 49) due to conceptual overlap
- Item 40 was removed because it got low score (I-CVI < 0.80) during round 2.Details of Item-level Content Validity Index (I-CVI) scores for each BeSD-HPV item in Delphi survey rounds 1 and 2 is shown in Table 1. S4 and S5 Files shows CVI scores of Delphi Round 1 and 2 on Excel Sheet respectively

**Table 1:** Item-level content validity index (I-CVI) scores for each BeSD-HPV item in Delphi survey rounds 1 and 2.

Original items	Round 1			Round 2		
	Relevance I-CVI	Clarity I-CVI	Consensus decision	Relevance I-CVI	Clarity I-CVI	Consensus decision
**How safe do you think HPV vaccine is for your daughter?**	1	1	A	1	1	A
**How much do you trust the health workers who give HPV vaccines?**	1	1	A	1	1	A
**To what extent do you agree that early screening and preventive measures, such as vaccination, are necessary to protect women from serious illnesses? (Not at all, To some extent, Neutral, To a considerable extent, To a great extent)**	1	0.9	R2	1	0.8	A
**Do you agree that vaccines administered in Pakistan could be used for experimental purposes? (Strongly disagree, Disagree, Neutral, Agree, strongly agree)**	0.8	0.9	R2	0.9	0.9	A
**Have you ever heard that vaccines may contain haram (prohibited) ingredients such as pork? (Not at all, To some extent, Neutral, To a considerable extent, To a great extent)**	0.9	0.9	R2	0.9	1	A
**To what extent do you think that the HPV vaccine beneficial to the women in Pakistan??(Not at all, To some extent, Neutral, To a considerable extent, To a great extent)**	0.8	0.8	R2	0.9	0.8	A
**To what extent do you believe that the HPV vaccine protects against cervical cancer? (Not at all, To some extent, Neutral, To a considerable extent, To a great extent)**	0.9	0.9	R2	1	1	A
**Do you feel you are fully informed about the benefits of the HPV vaccine? (Not at all, To some extent, Neutral, To a considerable extent, To a great extent)**	1	0.8	R2	1	1	A
**How much do you trust the HPV vaccine being introduced in your country? (Not at all, To some extent, Neutral, To a considerable extent, To a great extent)**	0.8	0.8	R2	0.9	0.9	A
**Do you think that if the government makes the HPV vaccine mandatory, some people will try to get a vaccination card without being vaccinated? (Strongly disagree, Disagree, Neutral, Agree, strongly agree)**	0.8	0.9	R2	0.9	1	A
**If the HPV vaccine were offered free of cost, do you think people would hesitate to receive it? (Not at all, To some extent, Neutral, To a considerable extent, To a great extent)**	0.8	0.9	R2	0.9	1	A
**Do you think the HPV vaccine could be part of efforts to control the population? (Strongly disagree, Disagree, Neutral, Agree, strongly agree)**	0.5	0.5	D	–	–	–
**Do you believe the HPV vaccine is acceptable in a Muslim society? (Strongly disagree, Disagree, Neutral, Agree, Strongly agree)**	0.8	0.8	R2	0.9	0.9	A
**To what extent do you agree that all girls aged 9–14 years should be vaccinated against HPV? (Strongly disagree, Disagree, Neutral, Agree, strongly agree)**	1	1	A	1	1	A
**If you believe your daughter is at low risk of cervical cancer, would you still get her vaccinated against HPV?(Certainly no, Probably no, Neutral, probably yes, certainly yes)**	0.8	0.8	R2	0.8	0.8	A
**Do you think the HPV vaccine will help protect your daughter from serious illness? (Strongly disagree, Disagree, Neutral, Agree, strongly agree)**	1	0.8	R2	1	0.9	A
**Do you think strong immunity reduces the importance of the HPV vaccine? (Not at all, To some extent, Neutral, To a considerable extent, To a great extent)**	0.8	0.8	R2	0.9	0.8	A
**To what extent are you concerned about the possible adverse effects of the HPV vaccine? (Not at all, To some extent, Neutral, To a considerable extent, To a great extent)**	0.9	0.9	A	0.9	0.9	A
**Do you think the HPV vaccine could negatively affect reproductive health? (Strongly disagree, Disagree, Neutral, Agree, strongly agree)**	0.9	0.9	A	0.9	0.9	A
**How much do you trust social media to get information about the HPV vaccine? (Strongly disagree, Disagree, Neutral, Agree, strongly agree)**	0.8	0.8	R2	0.9	0.9	A
**Do you think most parents you know would want you to get your daughter vaccinated against HPV?**	1	1	A	1	1	A
**Do you think most of your close family and friends want you to get your daughter vaccinated against HPV?**	1	1	A	1	1	A
**Has a health worker recommended your daughter vaccinated against HPV?**	1	1	A	1	1	A
**If it was time for your daughter to get vaccinated, would the mother need permission to take her to the clinic?**	1	1	A	1	1	A
**How often do you hear rumors or misconceptions about the HPV vaccine in your community? (Never, rarely. Neutral, Sometimes, Always)**	0.9	0.9	A	0.9	0.9	A
**To what extent do religious beliefs in your community influence decisions about the HPV vaccine? (Not at all, To some extent, Neutral, To a considerable extent, To a great extent)**	0.9	0.9	A	0.9	0.9	A
**Would people in your community trust information about the HPV vaccine available on social media? (Extremely unlikely, Unlikely, Neutral, Likely, extremely likely)**	0.8	0.8	A	0.8	0.8	A
**Do you think married and unmarried women may have different opinions about the HPV vaccine? (Strongly disagree, Disagree, Neutral, Agree, strongly agree)**	0.7	0.7	D	–	–	N/A
**Will you wait to observe its effects on others before deciding to get your daughter vaccinated? (Extremely unlikely, Unlikely, Neutral, Likely, extremely likely)**	0.7	0.8	R2	0.8	0.9	A
**If a trusted person in your community recommends the HPV vaccine, would you be more willing to get your daughter vaccinated? (Extremely unlikely, Unlikely, Neutral, Likely, extremely likely)**	0.9	0.9	R2	1	1	A
**Will you talk to your daughter about the HPV vaccine before deciding to get her vaccinated? (Extremely unlikely, Unlikely, Neutral, Likely, extremely likely)**	0.9	0.9	R2	1	1	A
**If you get your daughter vaccinated with the HPV vaccine, would you recommend it to others as well? (Extremely unlikely, Unlikely, Neutral, Likely, extremely likely)**	1	0.9	R2	1	1	A
**Do you prefer that the primary healthcare provider educating about the HPV vaccine be a female doctor? (Extremely unlikely, Unlikely, Neutral, Likely, extremely likely)**	1	1	R2	1	1	A
**Who do you trust most for accurate information about the HPV vaccine? (doctor, teacher, religious leader, community leader or elder, family member, friend)**	0.9	0.9	R2	1	1	A
**From which sources do you get information about the HPV vaccine? (religious leader, poster, healthcare worker, neighbor, social media, TV)**	0.8	0.8	R2	0.8	0.8	D
**Do you want your daughter to get vaccinated against HPV?**	0.9	0.9	R2	1	1	A
**When the HPV vaccine campaign begins, do you intend to register your daughter? (Extremely unlikely, Unlikely, Neutral, Likely, extremely likely)**	1	0.9	R2	1	1	A
**Do you believe vaccinating girls against HPV is as important as other childhood vaccines? (Strongly disagree, Disagree, Neutral, Agree, strongly agree)**	1	1	A	1	1	A
**If you know that cervical cancer cases are increasing among young women in your area, will it motivate you to get your daughter vaccinated? (Strongly disagree, Disagree, Neutral, Agree, strongly agree)**	0.9	0.8	R2	1	0.9	A
**If the government makes the HPV vaccine mandatory, will you get your daughter vaccinated? (Certainly no, probably no, Neutral, probably yes, certainly yes)**	0.8	0.9	R2	0.7	0.7	D
**If any of your children has ever experienced side effects from routine vaccination, would you still decide to get your daughter vaccinated with the HPV vaccine? (Extremely unlikely, Unlikely, Neutral, Likely, extremely likely)**	0.9	0.9	R2	1	1	A
**Compared to other vaccines, how much do you trust the safety of the HPV vaccine? (Not at all, To some extent, Neutral, To a considerable extent, To a great extent)**	1	1	A	1	1	A
**If you are given scientifically based information about the HPV vaccine, would you consider getting your daughter vaccinated? (Extremely unlikely, Unlikely, Neutral, Likely, extremely likely)**	0.9	0.9	R2	1	1	A
**If you learn that other countries are administering the HPV vaccine, would it increase your trust in vaccination? (Extremely unlikely, Unlikely, Neutral, Likely, extremely likely)**	1	1	A	1	1	A
**Do you want your daughter to get vaccinated against HPV?**	0.9	0.8	R2	0.7	0.7	D
**If hospital staff provide limited information about the HPV vaccine, will you still choose to get your daughter vaccinated? (Extremely unlikely, Unlikely, Neutral, Likely, extremely likely)**	0.9	0.9	R2	1	1	A
**If government offers monetary benefits for getting the HPV vaccine, would people be more willing to get vaccinated? (Strongly disagree, Disagree, Neutral, Agree, strongly agree)**	0.9	0.9	R2	1	1	A
**Do you trust that the HPV vaccine will be safely administered in schools? (Not at all, To some extent, Neutral, To a considerable extent, To a great extent)**	0.9	0.9	R2	1	1	A
**Do you think school administrations will actively support the HPV vaccination program? (Not at all, To some extent, Neutral, To a considerable extent, To a great extent)**	0.8	0.8	R2	0.8	0.8	D
**Do you agree that public and private schools have different attitudes towards vaccination campaigns? (Not at all, To some extent, Neutral, To a considerable extent, To a great extent)**	1	1	A	1	1	A
**Do you think teachers should be given specific training to raise awareness about the HPV vaccine among adolescent girls? (Strongly disagree, Disagree, Neutral, Agree, strongly agree)**	0.9	0.9	A	1	1	A
**Do you think girls should be given awareness about the HPV vaccine in schools? (Strongly disagree, Disagree, Neutral, Agree, strongly agree)**	0.9	0.9	R2	1	1	A
**Do you trust outreach vaccination services for your daughter’s HPV vaccination? (Not at all, To some extent, Neutral, To a considerable extent, To a great extent)**	0.9	0.9	R2	1	1	A
**Do you agree that people are concerned about the brand of the vaccine being used? (Not at all, To some extent, Neutral, To a considerable extent, To a great extent)**	1	1	R2	1	1	A
**Does the vaccination staff cooperate with you during the routine vaccination process? (Not at all, To some extent, Neutral, To a considerable extent, To a great extent)**	0.8	0.7	R2	0.9	0.8	A
**Where would you prefer your daughter to receive the HPV vaccine? (public hospital, private hospital, outreach service, school, others)**	0.9	0.9	A	1	1	A
**What barriers do you think exist in your community regarding access to vaccines? (long distance, lack of information, affordability issues, work or family responsibilities, unavailability of the vaccine, lack of transport, clinic timings)**	0.9	0.9	A	1	1	A
**How long do you usually wait at the health center for routine vaccination? (<10 min, 10–20 min, 21–30 min, > 30 min)**	0.9	0.9	R2	0.9	0.9	A
**Which social media platforms do you trust most for vaccine-related information? Select all that apply. (Tik-Tok, Facebook, Instagram, YouTube, Twitter, Other)**	0.9	0.9	A	1	1	A
**How helpful do you think reminders (e.g., mobile messages, school announcements) are to ensure timely vaccination? (Extremely unlikely, Unlikely, Neutral, Likely, extremely likely)**	1	1	A	1	1	A
**How often do you receive vaccine-related information through TV, radio or mobile phone? (Never, Rarely, Neutral, Sometimes, Always)**	1	1	A	1	1	A
**Who usually makes health decisions in your household? (Maternal grandparents, Paternal grandparents, Mother, Father, others)**	1	0.9	A	1	0.9	A
**Do you feel comfortable discussing reproductive health during family conversations? (Not at all, To some extent, Neutral, To a considerable extent, To a great extent)**	0.9	0.9	R2	1	1	A
**Do you think men should also be educated about the HPV vaccine through awareness campaigns? (Strongly disagree, Disagree, Neutral, Agree, strongly agree)**	1	1	A	1	1	A
**Would you choose to get your daughter vaccinated against HPV even if your family opposes it? (Extremely unlikely, Unlikely, Neutral, Likely, extremely likely)**	0.9	0.9	R2	1	1	A
**If your daughter wishes to receive the HPV vaccine but you have some reservations, how would you react? (Strongly oppose, Oppose, Neutral, Support, fully support)**	0.8	0.8	R2	0.8	0.9	A
**If people you know get their daughters vaccinated against HPV, would you be more willing to do the same? (Extremely unlikely, Unlikely, Neutral, Likely, extremely likely)**	0.9	0.8	D	–	–	N/A
**Do you think that hearing real stories of cervical cancer patients would increase parents’ trust in the HPV vaccine? (Extremely unlikely, Unlikely, Neutral, Likely, extremely likely)**	0.9	0.9	A	1	1	A
**If you see health care professionals vaccinating their own daughters against HPV, would you feel more confident about doing the same? (Extremely unlikely, Unlikely, Neutral, Likely, extremely likely)**	1	1	A	1	1	A
**Do you agree that a female vaccinator should administer the HPV vaccine to girls? (Strongly disagree, Disagree, Neutral, Agree, strongly agree)**	0.9	0.9	R2	1	1	A
**Do you agree that people in community hesitate to get the HPV vaccine due to social reasons? (Strongly disagree, Disagree, Neutral, Agree, strongly agree)**	0.9	0.9	R2	1	1	A
**Do you think it is appropriate to choose only girls for the HPV vaccine? (Strongly disagree, Disagree, Neutral, Agree, strongly agree)**	0.8	0.9	R2	0.9	1	A
**Do you think people from different ethnic backgrounds may hesitate to vaccinate their daughters against the HPV vaccine? (Not at all, To some extent, Neutral, To a considerable extent, To a great extent)**	0.9	0.9	R2	1	1	A
**Would you agree that the HPV vaccine should be administered to your daughter at school without your permission? (Not at all, To some extent, Neutral, To a considerable extent, To a great extent)**	0.8	0.8	R2	0.9	0.9	A

#### Interpretation.

The removal items that met numeric threshold (I-CVI > 0.80) indicate that experts go beyond the superficial scoring criteria based on numeric agreement and indicate deeper level of expert’s engagement to fully endorse emic perspective covering cultural nuance avoiding conceptual overlap and identified issue of redundancy that were not detected through etic numeric scoring.

### Final content validity assessment

The I-CVI and S-CVI were recalculated once the revised version was received from all experts. The scale-level CVI (S-CVI) score calculated after incorporating all changes was found to be 0.96. Details are shown in Tables 2 and 3.

**Table 2:** Content validity calculation for relevance.

Experts	1	2	3	4	5	6	7	8	9	10	Experts in agreement	I-CVI-Relevance	UA
Items	Relevance Ratings												
**1**	1	1	1	1	1	1	1	1	1	1	10	1	1
**2**	1	1	1	1	1	1	1	1	1	1	10	1	1
**3**	1	1	1	1	1	1	1	1	1	1	10	1	1
**4**	1	1	1	1	1	1	1	1	0	1	9	0.9	0
**5**	1	1	1	1	1	1	1	0	1	1	9	0.9	0
**6**	1	0	1	1	1	1	1	1	0	1	8	0.8	0
**7**	1	1	1	1	1	1	1	1	1	1	10	1	1
**8**	1	1	1	1	1	1	1	1	1	1	10	1	1
**9**	1	1	1	0	1	1	1	1	1	1	9	0.9	0
**10**	1	1	1	1	1	1	1	1	0	1	9	0.9	0
**11**	1	1	1	1	1	1	1	1	0	1	9	0.9	0
**12**	1	1	1	1	1	1	1	1	0	1	9	0.9	0
**13**	1	1	1	1	1	1	1	1	1	1	10	1	1
**14**	1	0	1	1	1	1	1	1	1	0	8	0.8	0
**15**	1	1	1	1	1	1	1	1	1	1	10	1	1
**16**	1	1	1	1	1	1	0	1	1	0	8	0.8	0
**17**	1	0	1	1	1	1	1	1	1	1	9	0.9	0
**18**	1	1	1	1	1	1	1	1	1	0	9	0.9	0
**19**	1	1	1	1	1	1	1	1	1	0	9	0.9	0
**20**	1	1	1	1	1	1	1	1	1	1	10	1	1
**21**	1	1	1	1	1	1	1	1	1	1	10	1	1
**22**	1	1	1	1	1	1	1	1	1	1	10	1	1
**23**	1	1	1	1	1	1	1	1	1	1	10	1	1
**24**	1	1	1	1	1	1	1	1	1	0	9	0.9	0
**25**	1	1	1	1	1	1	1	1	1	0	9	0.9	0
**26**	1	1	1	0	1	1	1	1	1	0	8	0.8	0
**27**	1	0	1	1	1	1	1	1	1	0	8	0.8	0
**28**	1	1	1	1	1	1	1	1	1	1	10	1	1
**29**	1	1	1	1	1	1	1	1	1	1	10	1	1
**30**	1	1	1	1	1	1	1	1	1	1	10	1	1
**31**	1	1	1	1	1	1	1	1	1	1	10	1	1
**32**	1	1	1	1	1	1	1	1	1	1	10	1	1
**33**	1	1	1	1	1	1	1	1	1	1	10	1	1
**34**	1	1	1	1	1	1	1	1	1	1	10	1	1
**35**	1	1	1	1	1	1	1	1	1	1	10	1	1
**36**	1	1	1	1	1	1	1	1	1	1	10	1	1
**37**	1	1	1	1	1	1	1	1	1	1	10	1	1
**38**	1	1	1	1	1	1	1	1	1	1	10	1	1
**39**	1	1	1	1	1	1	1	1	1	1	10	1	1
**40**	1	1	1	1	1	1	1	1	1	1	10	1	1
**41**	1	1	1	1	1	1	1	1	1	1	10	1	1
**42**	1	1	1	1	1	1	1	1	1	1	10	1	1
**43**	1	1	1	1	1	1	1	1	1	1	10	1	1
**44**	1	1	1	1	1	1	1	1	1	1	10	1	1
**45**	1	1	1	1	1	1	1	1	1	1	10	1	1
**46**	1	1	1	1	1	1	1	1	1	1	10	1	1
**47**	1	1	1	1	1	1	1	1	1	1	10	1	1
**48**	1	1	1	1	1	1	1	1	1	1	10	1	1
**49**	1	1	1	1	1	1	1	1	1	1	10	1	1
**50**	1	1	1	1	1	1	1	1	1	1	10	1	1
**51**	1	1	1	1	1	1	1	1	1	1	10	1	1
**52**	1	1	1	1	1	1	0	1	1	1	9	0.9	0
**53**	1	1	1	1	1	1	1	1	1	1	10	1	1
**54**	1	1	1	1	1	1	1	1	1	1	10	1	1
**55**	1	0	1	1	1	1	1	1	1	1	9	0.9	0
**56**	1	1	1	1	1	1	1	1	1	1	10	1	1
**57**	1	1	1	1	1	1	1	1	1	1	10	1	1
**58**	1	1	1	1	1	1	1	1	1	1	10	1	1
**59**	1	1	1	1	1	1	1	1	1	1	10	1	1
**60**	1	1	1	1	1	1	1	1	1	1	10	1	1
**61**	1	1	1	1	1	1	1	1	1	1	10	1	1
**62**	1	1	1	1	1	1	1	1	1	1	10	1	1
**63**	1	1	1	0	1	1	1	1	0	1	8	0.8	0
**64**	1	1	1	1	1	1	1	1	1	1	10	1	1
**65**	1	1	1	1	1	1	1	1	1	1	10	1	1
**66**	1	1	1	1	1	1	1	1	1	1	10	1	1
**67**	1	1	1	1	1	1	1	1	1	1	10	1	1
**68**	1	1	1	1	1	1	1	1	0	1	9	0.9	0
**69**	1	1	1	1	1	1	1	1	1	1	10	1	1
**70**	1	1	1	1	1	1	1	1	0	1	9	0.9	0
**Proportion Relevance**	1	0.92	1	0.95	1	1	0.97	0.98	0.88	0.88			
											Sum of I-CVI	67.3	
Average proportion of items judged as relevance across 10 experts											0.9614		
											S-CVI/Ave	0.9614	
											S-CVI/UA	0.7	

**Table 3:** Content validity calculation for clarity.

Experts	1	2	3	4	5	6	7	8	9	10	Experts in agreement	I-CVI-Clarity	UA
Items	Clarity Ratings												
**1**	1	1	1	1	1	1	1	1	1	1	10	1	1
**2**	1	1	1	1	1	1	1	1	1	1	10	1	1
**3**	1	0	1	1	1	1	1	0	1	1	8	0.8	0
**4**	1	0	1	1	1	1	1	1	1	1	9	0.9	0
**5**	1	1	1	1	1	1	1	1	1	1	10	1	1
**6**	1	1	1	1	1	1	1	0	0	1	8	0.8	0
**7**	1	1	1	1	1	1	1	1	1	1	10	1	1
**8**	1	1	1	1	1	1	1	1	1	1	10	1	1
**9**	1	1	1	0	1	1	1	1	1	1	9	0.9	0
**10**	1	1	1	1	1	1	1	1	1	1	10	1	1
**11**	1	1	1	1	1	1	1	1	1	1	10	1	1
**12**	1	1	1	1	1	1	1	1	0	1	9	0.9	0
**13**	1	1	1	1	1	1	1	1	1	1	10	1	1
**14**	1	0	1	1	1	1	1	1	1	0	8	0.8	0
**15**	1	1	1	1	1	1	1	1	0	1	9	0.9	0
**16**	1	1	1	1	1	1	0	1	1	0	8	0.8	0
**17**	1	1	1	1	1	1	1	1	1	0	9	0.9	0
**18**	1	1	1	1	1	1	1	1	1	0	9	0.9	0
**19**	1	1	1	1	1	1	1	1	1	0	9	0.9	0
**20**	1	1	1	1	1	1	1	1	1	1	10	1	1
**21**	1	1	1	1	1	1	1	1	1	1	10	1	1
**22**	1	1	1	1	1	1	1	1	1	1	10	1	1
**23**	1	1	1	1	1	1	1	1	1	1	10	1	1
**24**	1	1	1	1	1	1	1	1	1	0	9	0.9	0
**25**	1	1	1	1	1	1	1	1	1	0	9	0.9	0
**26**	1	1	1	0	1	1	1	1	1	0	8	0.8	0
**27**	1	1	1	1	1	1	1	1	1	0	9	0.9	0
**28**	1	1	1	1	1	1	1	1	1	1	10	1	1
**29**	1	1	1	1	1	1	1	1	1	1	10	1	1
**30**	1	1	1	1	1	1	1	1	1	1	10	1	1
**31**	1	1	1	1	1	1	1	1	1	1	10	1	1
**32**	1	1	1	1	1	1	1	1	1	1	10	1	1
**33**	1	1	1	1	1	1	1	1	1	1	10	1	1
**34**	1	1	1	1	1	1	1	1	1	1	10	1	1
**35**	1	1	1	1	1	1	1	1	1	1	10	1	1
**36**	1	1	1	1	1	1	1	0	1	1	9	0.9	0
**37**	1	1	1	1	1	1	1	1	1	1	10	1	1
**38**	1	1	1	1	1	1	1	1	1	1	10	1	1
**39**	1	1	1	1	1	1	1	1	1	1	10	1	1
**40**	1	1	1	1	1	1	1	1	1	1	10	1	1
**41**	1	1	1	1	1	1	1	1	1	1	10	1	1
**42**	1	1	1	1	1	1	1	1	1	1	10	1	1
**43**	1	1	1	1	1	1	1	1	1	1	10	1	1
**44**	1	1	1	1	1	1	1	1	1	1	10	1	1
**45**	1	1	1	1	1	1	1	1	1	1	10	1	1
**46**	1	1	1	1	1	1	1	1	1	1	10	1	1
**47**	1	1	1	1	1	1	1	1	1	1	10	1	1
**48**	1	1	1	1	1	1	1	1	1	1	10	1	1
**49**	1	1	1	1	1	1	1	1	1	1	10	1	1
**50**	1	1	1	1	1	1	1	1	1	1	10	1	1
**51**	1	1	1	1	1	1	1	1	1	1	10	1	1
**52**	1	1	1	1	1	1	0	0	1	1	8	0.8	0
**53**	1	1	1	1	1	1	1	1	1	1	10	1	1
**54**	1	1	1	1	1	1	1	1	1	1	10	1	1
**55**	1	0	1	1	1	1	1	1	1	1	9	0.9	0
**56**	1	1	1	1	1	1	1	1	1	1	10	1	1
**57**	1	1	1	1	1	1	1	1	1	1	10	1	1
**58**	1	1	1	1	1	1	1	1	1	1	10	1	1
**59**	1	1	1	1	1	1	1	1	0	1	9	0.9	0
**60**	1	1	1	1	1	1	1	1	1	1	10	1	1
**61**	1	1	1	1	1	1	1	1	1	1	10	1	1
**62**	1	1	1	1	1	1	1	1	1	1	10	1	1
**63**	1	1	1	0	1	1	1	1	1	1	9	0.9	0
**64**	1	1	1	1	1	1	1	1	1	1	10	1	1
**65**	1	1	1	1	1	1	1	1	1	1	10	1	1
**66**	1	1	1	1	1	1	1	1	1	1	10	1	1
**67**	1	1	1	1	1	1	1	1	1	1	10	1	1
**68**	1	1	1	1	1	1	1	1	1	1	10	1	1
**69**	1	1	1	1	1	1	1	1	1	1	10	1	1
**70**	1	1	1	1	1	1	1	1	0	1	9	0.9	0
**Proportion Clarity**	1	0.94	1	0.95	1	1	0.97	0.94	0.92	0.87			
											Sum of I-CVI	67.3	
Average proportion of items judged as clarity across 10 experts													
											S-CVI/Ave	0.9614	
											S-CVI/UA	0.7	

**English version of 70 item questionnaire BeSD HPV BT1 & T2 has been attached as S7 File.**

#### Interpretation.

The substantial improvement in S-CVI from 0.88 to 0.96 demonstrates a significant improvement in overall content validity, confirming that iterative revision strengthened item clarity, reduced redundancy, and improved cultural appropriateness. The strengthened scale-level validity indicates that the adapted BESD HPV TOOL more accurately captures behavioral and social drivers of HPV vaccination in the Pakistani context.

This entire process ensured robustness of process by triangulation of numeric findings with culturally grounded qualitative reflection thus ensuring strong cultural relevance.

Excel sheet for Content Validation and CVI scores has been uploaded as S6 File.

### Stage 2: Response process validity

**1. Pilot Testing (cognitive Interviews)**

After two rounds of Delphi, the final synthesized and reviewed version of the BeSD-HPV tool was pilot-tested with caregivers/parents of potential vaccine recipients (girls between 9–16 years) through cognitive interviews.

**2. Rationale**

This phase focuses on:

- Capturing a variety of responses from potential caregivers/parents based on individual attributes such as age, gender, education, and parental role in HPV decision making
- Fixing the issues of comprehension and clarity of items
- Recognizing problematic phrases, language, analyzing sensitive concepts and misunderstanding among diverse participants
- Iteratively improve and refine items until no new cognitive problems emerge (informal saturation).

**3. Participants**

Thirty parents/caregivers were selected via convenience sampling as per standard guidelines for cognitive interviewing. Data was collected through interviews with parents/potential caregivers of adolescents (aged 9–16 years). Data was collected from potential participants after they provided informed consent.

**4. Sample size justification & Sampling techniques**

Since the goal of cognitive interview process is to ensure interpretability and cultural relevance, a small purposively selected representative sample is methodologically suitable and also consistent with international guidelines.The objective of this phase was to further improve clarity, comprehension and cultural appropriateness, hence a purposive sample comprising of parents having varying sociodemographic characteristics was selected to ensure variability and to get depth of responses. A total of 30 parents were recruited from both Northern and Southern Punjab, representing broad age groups, income levels and geographical location. Participants recruited were within age group ranging between 30–50 years, belonging from diverse socioeconomic strata and income levels with varied occupation background including both educated and educated individuals. This heterogeneity ensured capturing of wide range of cultural perspectives, thereby establishing representativeness and ensuring robustness of cognitive interview process.

**5. Techniques**

Cognitive interviews with caregivers from varied literacy and socio-cultural backgrounds used think-aloud and probing techniques.

a **Probing method**

During the probing method participants were asked how they interpreted each item, why they chose a specific response, and if any phrases or wording were confusing or inappropriate. This method helps identify idiomatic and semantic issues, ensuring that every item is interpreted as intended.

b **Think-aloud technique**

In the think-aloud technique participants were asked to verbalize their thoughts as they answered each question. This process provides insight into judgment and cognitive processing behind their responses. This process further enhanced conceptual and experiential equivalence. This procedure ensures that all items are relevant to the sociocultural context of Pakistan.

**6. Cognitive Interview Analysis**

Cognitive interviews were analysed using a structured categorization approach, in which two independent researchers classified each item into predefined categories based on participant’s judgment, comprehension, retrieval and response processes. To enhance analytic rigor initial analysis was carried out by both researchers independently followed by comparative analysis in which both researchers compared their categories and discussed discrepancies by referring back to transcripts and field notes to ensure interpretations were grounded in participant’s responses. In case in disagreement, a third senior researcher (SN) reviewed the items and provided adjudication. Final consensus was achieved through reflective deliberation and iterative discussion.

Consensus on categorization was reached between two independent researchers Items were categorized into 3 categories:

(1) items with no problems in understanding
(2) items with minor problems in understanding
(3) items with major problems in understanding

Seventeen items requiring clarification were revised and re-worded after review by all authors. Details are shown in Table 4.

**Table 4:** Cognitive interviews: Issues identified, and revisions done.

Item No.	Items	Issues categorized	Rephrased version
15	In your opinion, does strong immunity influence the decision to receive HPV vaccine? (Strongly disagree, Disagree, Neutral, Agree, strongly agree)	Response scale inconsistency	To what extent do you think a person’s strong natural immunity affects their decision to receive the HPV vaccine (Not at all, To some extent, Neutral, To a considerable extent, To a great extent)
17	Do you think the HPV vaccine could negatively affect reproductive health? (Strongly disagree, Disagree, Neutral, Agree, strongly agree)	Response scale inconsistency	To what extent do you think the HPV vaccine could negatively affect reproductive health? (Not at all, To some extent, Neutral, To a considerable extent, To a great extent)
19	How often do you hear rumors or misconceptions about the HPV vaccine in your community? (Never, rarely. Neutral, Sometimes, Always)	Response scale inconsistency	How often do you hear rumors or misconceptions about the HPV vaccine in your community? (Not at all, To some extent, Neutral, To a considerable extent, To a great extent)
21	Would people in your community trust information about the HPV vaccine available on social media? (Extremely unlikely, Unlikely, Neutral, Likely, extremely likely)	Response scale inconsistency	To what extent do people in your community trust information about the HPV vaccine shared on social media platforms? (Not at all, To some extent, Neutral, To a considerable extent, To a great extent)
27	Who do you trust most for accurate information about the HPV vaccine? (a healthcare provider, community leader, religious leader, elders in the family, media)	Limited detail due to single-response format/ inability to capture degrees of trust across multiple sources	To what extent do you trust the following sources for accurate information about the HPV vaccine? (Not at all, to some extent, Neutral, to a considerable extent) Each source to be largely rated individually* (a healthcare provider, community leader, religious leader, elders in the family, media)
28	From which sources do you get information about the HPV vaccine? (Healthcare provider, Community leader, religious leader, Elders in the family, Media)	Original items don’t compare the relative importance of one source versus another.	To what extent do you rely on the following sources for information about the HPV vaccine? (Not at all, to some extent, Neutral, to a considerable extent) Each source to be largely rated individually*
30	Do you believe vaccinating girls against HPV is as important as other childhood vaccines? (Strongly disagree, Disagree, Neutral, Agree, strongly agree)	Response scale inconsistency	To what extent do you believe vaccinating girls against HPV is as important as receiving other childhood vaccines? (Not at all, To some extent, Neutral, To a considerable extent, To a great extent)
41	Do you think the implementation of mandatory legislation would increase support for the HPV vaccination program? (Not at all, To some extent, Neutral, To a considerable extent, To a great extent)	Minor problem in understanding	If HPV vaccination becomes a legal requirement (e.g., for school admission or travel), would you ensure that the girls in your family receive the vaccine? (Not at all, To some extent, Neutral, To a considerable extent, To a great extent)
43	Do you think teachers should be given specific training to raise awareness about the HPV vaccine among adolescent girls? (Strongly disagree, Disagree, Neutral, Agree, strongly agree)	Response scale inconsistency	To what extent do you think teachers should receive specific training to raise awareness about the HPV vaccine among adolescent girls? (Not at all, To some extent, Neutral, To a considerable extent, To a great extent)
48	Where would you prefer the HPV vaccination to be given? (1. School. 2.Fixed site 3. outreach)	Original item restricts preferences to one site and does not capture degree of preference	To what extent would you prefer each of the following places as the site for receiving the HPV vaccination? (Not at all, to some extent, Neutral, to a considerable extent) Each site to be largely rated individually* 1. School. 2.Fixed site3. outreach
49	What barriers do you think exist in your community regarding access to vaccines? (Long distance, lack of information, affordability issues, work or family responsibilities, unavailability of the vaccine, lack of transport, clinic timings)	Original item only identifies barriers without capturing their severity or extent	To what extent do you think each of the following barriers exists in your community regarding access to vaccines? (Not at all, to some extent, Neutral, to a considerable extent) Each barrier to be primarily rated individually*
50	How long do you usually wait at the health center for routine vaccination? (<10 min, 10–20 min, 21–30 min, >30 min)	Response scale inconsistency	To what extent do you usually experience long waiting times at the health center for routine vaccination? (Not at all, To some extent, Neutral, To a considerable extent, To a great extent
51	Which social media platforms do you trust most for vaccine-related information? Select all that apply. (Tik-Tok, Facebook, Instagram, YouTube, Twitter, Other)	Inability to capture degrees of trust across multiple sources	To what extent do you trust each of the following social media platforms for vaccine-related information? (Not at all, To some extent, Neutral, To a considerable extent, To a great extent) **Platforms to be rated separately:** • TikTok • Facebook • Instagram • YouTube • Twitter/X • Other (please specify)
52	How helpful do you think reminders (e.g., mobile messages, school announcements) are to ensure timely vaccination? (Extremely unlikely, Unlikely, Neutral, Likely, extremely likely)	Response scale inconsistency	To what extent do you think reminders (e.g., mobile messages, school announcements) are helpful in ensuring timely vaccination? (Not at all, To some extent, Neutral, To a considerable extent, To a great extent)
53	How often do you receive vaccine-related information through TV, radio or mobile phone? (Never, rarely. Neutral, Sometimes, Always)	Response scale inconsistency	To what extent do you receive vaccine-related information through TV, radio, or mobile phone? (Not at all, To some extent, Neutral, To a considerable extent, To a great extent)
54	Who usually makes health decisions in your household? (Maternal grandparents, Paternal grandparents, Mother, Father, others)	Limited detail due to single-response format/ inability to capture degrees of influence across multiple sources	To what extent do each of the following usually make health decisions in your household? (Not at all, To some extent, Neutral, To a considerable extent, To a great extent) (Maternal grandparents, Paternal grandparents, Mother, Father, others)
56	Do you think men should also be educated about the HPV vaccine through awareness campaigns? (Strongly disagree, Disagree, Neutral, Agree, strongly agree)	Response scale inconsistency	To what extent do you think men should be educated about the HPV vaccine through awareness campaigns? (Not at all, To some extent, Neutral, To a considerable extent, To a great extent)
59	Do you think that hearing real stories of cervical cancer patients would increase parents’/caregivers trust in the HPV vaccine? (Extremely unlikely, Unlikely, Neutral, Likely, extremely likely)	Response scale inconsistency	To what extent do you think that hearing real stories of cervical cancer patients would increase parents’/caregivers’ trust in the HPV vaccine? (Not at all, To some extent, Neutral, To a considerable extent, To a great extent)

**7. Documentation**

All translation decisions, justifications for changes, references to supporting evidence, and data obtained from qualitative sources along with and their rationale, were documented in an extensive adaptation log. Reproducibility and Transparency were ensured through this documentation, while also maintaining the ability to compare the BeSD tool adapted with that of other countries.

**8. Reporting Guidelines:**

This study employed a Delphi technique to achieve consensus among experts, following established guidelines for conducting and reporting Delphi studies (CREDES). The research instrument underwent cross-cultural adaptation using Beaton’s guidelines, which involved forward and backward translation, synthesis, and expert review to ensure conceptual, linguistic, and cultural equivalence. The adapted instrument was pilot tested with a small group of participants to ensure clarity and relevance. The Delphi process involved multiple rounds of questionnaires, with feedback provided to panel members after each round. Data analysis and reporting followed the CREDES guidelines to ensure transparency and rigor [1,17].

### Response process validity

30 parents/caregivers participated in cognitive interviews using a think-aloud technique and probing. Seventeen items categorized as having minor or major issues were rephrased by the authors based on participants’ feedback, for better comprehension (Refer to Table IV).

**Fig 2:**
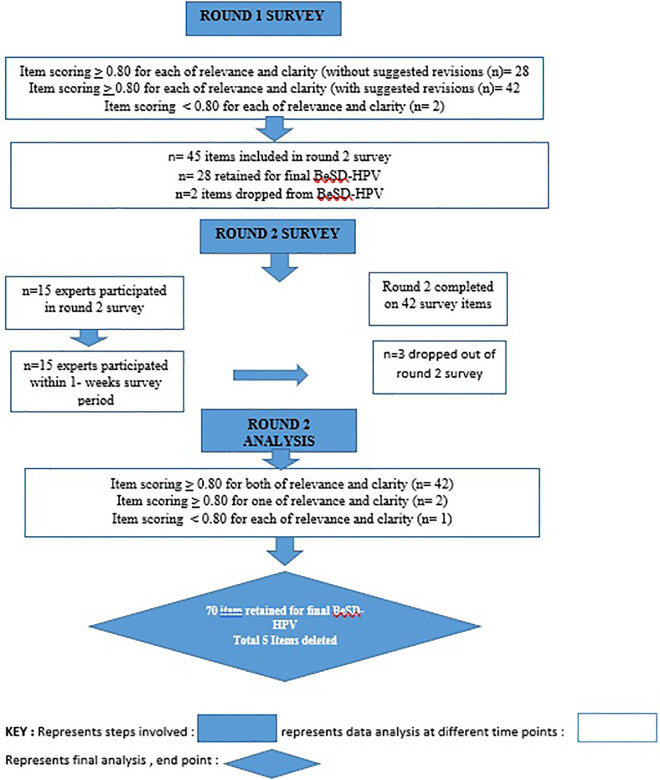
Stepwise flow of item reduction, revision, and consensus achievement during Delphi rounds.

Fig 2 presents the stepwise flow of item reduction, revision, and consensus achievement during Delphi rounds.

## Discussion

The present study rigorously adapted the WHO Behavioral and Social Drivers (BeSD) framework for HPV vaccination within the Pakistani context, following established cross-cultural adaptation methodologies and guided by emic–etic theoretical principles. By applying the six-step process outlined in Beaton et al. (2000) and reinforced by WHO guidelines (2016), we ensured semantic, idiomatic, experiential, and conceptual equivalence of items while embedding culturally specific drivers of vaccination decision-making [11].

While the original BeSD framework provided a robust global structure for understanding behavioral and social drivers of vaccination, our findings highlight that etic foundations did not fully capture the culturally embedded influences shaping HPV vaccine acceptance in Pakistan. The emic insights generated through qualitative work and expert review revealed dimensions of decision-making that were not fully represented in the original framework, resulting in significant refinement and extension of BeSD domains.

Our findings supported the notion that following this rigorous methodology, the adapted BeSD-HPV tool was able to retain cross-cultural comparability while also ensuring enriched contextual relevance by including emically derived new items, such as patriarchal hierarchies, power dynamics within family systems, and cultural norms that influence vaccination decision making. In general, the term “adaptation” is employed to evaluate a phenomenon across cultural boundaries [18]. It includes a series of flexible steps that permit the components of a tool or questionnaire to change while preserving the fundamental framework, functionality, and structure [19].

Researchers generally agree that there are two distinct ways to approach cross-cultural adaptation with the “emic etic paradigm”. The phenomena under investigation, from the “emic” perspective, identify the intra-cultural components “from the inside”, their evaluation within the culture and its surroundings. The evaluation of the phenomenon from “outside” the culture is part of the “etic” perspective, which compares other cultures [20].

There is a common consensus among researchers that cross- cultural adaptation can be approached from two different domains, the “emic etic paradigm” [21]. The “emic” perspective of the phenomenon under study indicates the intra-cultural elements “from the inside”, their assessment within the culture and its context. The “etic” perspective involves comparing across different cultures, as it entails assessing the phenomenon from “outside” the culture [4]. Each of these viewpoints has unique benefits and drawbacks. Although the “emic” approach provides a thorough comprehension of the culture, it is not generalizable, in contrast to the “etic” approach, in which reliability is prioritized over the validity of the research findings [20].

The primary goal of adaptation is to make an instrument relevant to a culture distinct from the one for which it was originally created [22]. According to published data, cross-cultural studies have steadily increased over the past 20 years and resulted in the adaptation and validation of culturally sensitive tools and instruments [23–25].

Research indicates that, although certain test adaptation criteria have been proposed, there is a lack of consensus on best practices or minimal adaptation standards [26]. Since the adaptation studies are difficult to conduct in low- and middle-income countries because of a lack of resources, there hasn’t been much research to emphasize these factors [23]. Depending on the goals for a given group of people, the adaptation of tools or instruments can range from superficial to deep processes, ranging from covering only language translation to addressing deeper phenomena, including the use of culturally relevant illustrations to support ethnographic research that incorporates culturally held values and idioms [27].

In our study, the integration of emic and etic approaches was crucial to maintain a balance between the necessity of global standards and local cultural sensitivity. The structural and theoretical foundation was supported by the imposed etic starting point, the original BeSD tool, thus ensuring the alignment with international literature on vaccination behavioral science. Contextual determinants and cultural discrepancies were incorporated through emic exploration, which was achieved through qualitative investigation and expert consensus that were not adequately captured in the original BeSD framework. A fundamental contribution of emic viewpoint is its elaboration of gendered power dynamics that strongly influence the vaccine decision making in patriarchal society. The original BeSD framework covers influence of social processes, but it does not explicitly include deeply embedded normative prospects regarding gender role in South Asian context. The intrahousehold hierarchy is strongly influenced by gendered decision system where vaccine decision is not attributed solely to individual attitude rather it’s a complex phenomenon strongly influenced by patriarchal and multigenerational household structures. These culturally grounded items strongly influence the sociocultural relevance of adapted tools. Influence of religious perspective also emerged as emically derived as determinant where many caregivers’ belief that endorsement of religious leaders is mandatory for vaccine decision making specifically considering it as medical intervention linked to adolescent sexual health. Another domain where emic insights challenged the original framework was the area of thinking and feeling where strong resentment was observed due to fear of side effects. Many parents were hesitant because of reproductive side effects and incapability to become pregnant in future. Concerns about Hilal ingredients was another major concern that was captured emically. Emic insights also influenced the motivation and practical issues domains by highlighting the culturally embedded obstacles like restricted female mobility, reluctance in discussing issues related to female reproductive health, need for female vaccinator for vaccination. Moreover, resistance was also associated with perceived unnecessity of vaccination and lack of sufficient scientific evidence to build trust for vaccine uptake.

The domain where new concepts emerged and emic insights challenged the original BeSD framework was cultural integration domain. In contrast to high income countries where health decision making is governed by individual autonomy, while in Pakistan vaccine uptake behavior is strongly influenced by social legitimacy and collective decision of intra generational household hierarchies.

Finally, synthesis of these perspectives resulted in a derived etic version of the BeSD (HPV-BeSD), which was not only aligned with global standards but also encompassed the culturally embedded construct pertinent to the sociocultural context of Pakistan. This bit-by-bit integration aligns with theoretical models of cross-cultural research that emphasize the complementarity of emic and etic approaches [28,29].

Our findings align with prior studies that highlight the importance and necessity of culturally adapting vaccination-related tools in LMICs settings. Harry et al. pointed out the importance of incorporating the influence of religious and community leaders into HIV prevention tools in Aba, Nigeria, a contextually parallel finding to our inclusion of religious endorsement in HPV vaccine adaptation [30]. Similarly, Alansari et al. (2018), in adapting the vaccination attitudes examination (VAX) scale, reported that mere translation alone failed to encapsulate culturally specific dimensions, emphasizing the need for item modification and adaptation [31]. Similarly, a Chinese study on assessing vaccine hesitancy using the WHO scale for caregivers of children under 3 showed that experiential equivalence was gained only after pretesting items with local caregivers to assess resonance with lived realities [32]. These findings conjointly emphasize that translation without cultural adaptation risks losing conceptual fidelity.

The strength of our study lies in its the unique approach, which differs from much published research on cultural adaptation by explicitly integrating the emic–etic theoretical framework as a guiding principle throughout the process. While many studies follow procedural adaptation steps (translation, back-translation, expert review), hardly any have actually highlighted a strong theoretical rationale for when and why new items should be added [23]. Our study illustrates that emic insights are not simply complementary but crucial for validity, especially in sociocultural contexts where vaccine decision-making is shaped by collective authority, religious norms, and gendered roles, factors less prominent in high-income country settings where much of the original validation work was performed. Consequently, the items that undergo significant modification, deletion, or addition were accurately identified through emic exploration. Moreover, the methodological rigor achieved through Delphi rounds further emphasizes the value of blending cultural theory with a robust methodological approach.

The overall content validity indices (I-CVI and S-CVI/Ave) acquired in this study further support the robustness of the adapted tool. With an S-CVI/Ave above 0.90, the adapted BeSD illustrates excellent content validity, consistent with standards guidelines for content validation [33–35]. In spite of these strengths, some limitations warrant consideration. Our adaptation was held in selected districts of Pakistan and may not fully enlighten divergence across provinces or ethnic groups. Future studies should reproduce the adaptation and validation process in other regions to ensure comprehensive generalizability. In addition, while our rigorous process emphasized semantic and experiential equivalence, psychometric testing of the adapted tool in larger representative samples remains a critical next step to establish reliability and construct validity. The next phase will focus on full psychometric validation including establishing construct validity by performing factor analysis to confirm domain structure, reliability analysis to establish internal consistency and test-retest reliability.

Large-scale field testing in diverse geographic settings will also be important to ensure robustness and generalizability beyond the study districts. Once validated, the adapted HPV-BeSD can be institutionalized as a contextually grounded assessment framework to guide HPV vaccine introduction, scale-up, and communication strategies across Pakistan.

## Conclusion

In conclusion, our study highlights the importance of combining systematic cross-cultural adaptation methods with emic–etic theoretical foundations when adapting global behavioral frameworks, such as BeSD, to specific cultural contexts. This dual approach not only retains comparability across settings but also ensures contextual sensitivity, enriching the tool’s ability to inform policy and interventions for HPV vaccination uptake in Pakistan and potentially other LMICs.

Several actionable implications guide the translation of these findings into practice. First, the theory-driven cultural adaptation of the WHO BeSD framework for HPV vaccination, can serve as evidence-based instrument for program managers and policy makers to diagnose behavior and social drivers of vaccine hesitancy at community level and to design effective targeted interventions to support the vaccine uptake during phase 2 of HPV vaccine roll out. Second incorporating emic perspectives including cultural integration can also serve as road map aligned with contextual realism to enhance vaccine acceptability. Thirdly, incorporation of this framework into routine immunization planning can guide all relevant stakeholders including representatives from country office of UN agencies, policy makers, front line healthcare workers, and immunization programs lead to aligning their efforts to support the uptake in alignment with evidence-based strategies.The next phase of research will focus on psychometric validation, including confirmatory factor analysis to establish construct validity and reliability testing in larger, representative samples.

## Supporting information

S1 File 6307959999163931338.
(PDF)

S2 File 6308779276111738687.
(PDF)

S3 File 6309108558192286196.
(PDF)

S4 File 6309880716623591134.
(PDF)

S5 File 6309900045229294736.
(PDF)

S6 File 6310643934311024570.
(PDF)

S7 File 6312380917129317419 (1).
(PDF)

S8 File 6312380917129317419.
(PDF)

S9 File 6312471961062298108 (1).
(PDF)

S10 File 6312471961062298108.
(PDF)

S11 File 6313051788797179737 (1).
(PDF)

S12 File Cognitive interview HPV_21_Nov (1).
(XLSX)

S13 File Supplementary file 4. CVI_file_20_Aug_Delphi_round_1&2.
(XLSX)
