# Supplementary material for: Cultural adaptation and content validation of the WHO BeSD framework for HPV vaccination in Pakistan: A two-phase Delphi and cognitive interview study
Source: PLoS One. 2026 Feb 17;21(2):e0335358. doi: 10.1371/journal.pone.0335358 (PMC12912614; doi:10.1371/journal.pone.0335358)
Supplement: S3 File — 6309108558192286196. (PDF) [file pone.0335358.s003.pdf]

Thinking & Feeling

	How relevant is this item	How clear is this item	Suggestons (if any)
1. To what extent do you agree that early screening and preventive measures, such as vaccination, are necessary to protect women from serious illnesses?<br>(Not at all, To some extent, Neutral, To a considerable extent, To a great extent)	4 (very relevant)	3 (clear but needs minor revisi on)	
2. Do you agree that vaccines administered in Pakistan could be used for experimental purposes?<br>(Strongly disagree, Disagree, Neutral, Agree, Strongly agree)	2 (item needs some revision)	1 (not clear)	This is a dichotomous question, Yes I agree, no I don't as there is a lot of grey involved in this statement.
3. **Have you ever heard that vaccines may contain haram (prohibited) ingredients such as pork?**<br>(Not at all, To some extent, Neutral, To a considerable extent, To a great extent)	1 (not relevant)	1 (not clear)	wouldn't this be an issue for muslim men and women only? It cannot be generalized to the entire population of Pakistan.
4. Do you think the HPV vaccine is being introduced unnecessarily?(Not at all, To some extent, Neutral, To a considerable extent, To a great extent)	2 (item needs some revision)	1 (not clear)	Again a dichotomous question. My suggestion...To what extent is the HPV vaccine beneficial to women in Pakistan?
5. To what extent do you believe that the HPV vaccine protects against cervical cancer?<br>(Not at all, To some extent, Neutral, To a considerable extent, To a great extent)	2 (item needs some revision)	1 (not clear)	It all depends upon my baseline knowledge. Have we asked the participants regarding this?
6. Do you feel you are fully informed about the benefits of the HPV vaccine?<br>(Not at all, To some extent, Neutral, To a considerable extent, To a great extent)	3 (relevant but needs minor revision)	2 (item needs some revisi on)	
7. How much do you trust the HPV vaccine being introduced in your country?<br>(Not at all, To some extent,	2 (item needs some revision)	2 (item needs some	Trust is a very subjective feeling. How can you quantify that?

	How relevant is this item	How clear is this item	Suggestions (if any)
Neutral, To a considerable extent, To a great extent)		revision)	
8. Do you think that if the government makes the HPV vaccine mandatory, some people will try to get a vaccination card without actually being vaccinated? (Strongly disagree, Disagree, Neutral, Agree, Strongly agree)	2 (item needs some revision)	2 (item needs some revision)	People will get a card due to their ill-informed notions or biases. This can be added to the statement
9. If the HPV vaccine were offered free of cost, do you think people would hesitate to receive it? (Not at all hesitant, somewhat hesitant, Neutral, Quite hesitant, Extremely hesitant)	2 (item needs some revision)	2 (item needs some revision)	IF HPV were made a part of the EPI program?
10. Do you think the HPV vaccine could be part of efforts to control the population? (Strongly disagree, Disagree, Neutral, Agree, Strongly agree)	2 (item needs some revision)	2 (item needs some revision)	Very direct question asking for information I might not be very comfirtable in sharing.
11. Do you believe the HPV vaccine is acceptable in a Muslim society? (Strongly disagree, Disagree, Neutral, Agree, Strongly agree)	2 (item needs some revision)	2 (item needs some revision)	Controversial question. would recommend rephrasing
12. To what extent do you agree that all girls aged 9 to 14 years should be vaccinated against HPV?(Strongly disagree, Disagree, Neutral, Agree, Strongly agree)	4 (very relevant)	4 (very)clear	
13. If you believe your daughter is at low risk of cervical cancer, would you still get her vaccinated against HPV?(Certainly no, Probably no, Neutral, Probably yes, Certainly yes)	4 (very relevant)	2 (item needs some revision)	Good question but is again very subjective.
14. Do you think the HPV vaccine will help protect your daughter from serious illness? (Strongly disagree, Disagree, Neutral, Agree, Strongly agree)	4 (very relevant)	2 (item needs some revision)	Why only my daughter, why not the women in my family?
15. **Do you think strong immunity reduces the importance of the HPV vaccine?**(Not at all, To some extent, Neutral, To a	2 (item needs some revision)	2 (item needs some revision)	Rather than strong immunity, it is the reduction of risk factors that effects this decision.

	How relevant is this item	How clear is this item	Suggestions (if any)
considerable extent, To a great extent)			
16. **To what extent are you concerned about** the **possible** adverse effects of the HPV vaccine? (Not at all, To some extent, Neutral, To a considerable extent, To a great extent)	4 (very relevant)	4 (very)clear	
17. Do you think the HPV vaccine could negatively affect reproductive health? (Strongly disagree, Disagree, Neutral, Agree, Strongly agree)	4 (very relevant)	4 (very)clear	
18. How much do you trust social media to get information about the HPV vaccine? (Strongly disagree, Disagree, Neutral, Agree, Strongly agree)	2 (item needs some revision)	2 (item needs some revision)	The scale does not match the item. If you are asking about my trust in social media, then it should be Not at all, To some extent, Neutral, To a considerable extent, To a great extent

Social Processes

	How relevant is this item	How clear is this item	Suggestions (if any)
19. How often do you hear rumors or misconceptions about the HPV vaccine in your community? (Never, Rarely. Neutral, Sometimes, Always)	4 (very relevant)	4 (very clear)	
20. To what extent do religious beliefs in your community influence decisions about the HPV vaccine? (Not at all, To some extent, Neutral, To a considerable extent, To a great extent)	4 (very relevant)	4 (very clear)	
21. Would people in your community trust information about the HPV vaccine available on social media? (Extremely unlikely, Unlikely, Neutral, Likely, Extremely likely)	4 (very relevant)	4 (very clear)	
22. Do you think married and unmarried women may have different opinions about the HPV vaccine? (Strongly disagree, Disagree,	4 (very relevant)	4 (very clear)	

	How relevant is this item	How clear is this item	Suggestions (if any)
Neutral, Agree, Strongly agree)			
23. Will you wait to observe its effects on others before deciding to get your daughter vaccinated?(Extremely unlikely, Unlikely, Neutral, Likely, Extremely likely)	2 (item needs some revision)	1 (not clear)	Item should not have terms like its effects...the effects of HPV vaccine and I would not use the term daughter
24. If a trusted person in your community recommends the HPV vaccine, would you be more willing to get your daughter vaccinated?(Extremely unlikely, Unlikely, Neutral, Likely, Extremely likely)	2 (item needs some revision)	2 (item needs some revision)	define trusted and replace the term daughter
25. Will you talk to your daughter about the HPV vaccine before deciding to get her vaccinated?(Extremely unlikely, Unlikely, Neutral, Likely, Extremely likely)	2 (item needs some revision)	2 (item needs some revision)	This question may be culturally inappropriate for men
26. If you get your daughter vaccinated with the HPV vaccine, would you recommend it to others as well?(Extremely unlikely, Unlikely, Neutral, Likely, Extremely likely)	4 (very relevant)	2 (item needs some revision)	would recommend replacing daughter
27. Do you prefer that the primary healthcare provider educating about the HPV vaccine be a female doctor?(Extremely unlikely, Unlikely, Neutral, Likely, Extremely likely)	4 (very relevant)	4 (very clear)	needs the grammer to be improved
28. Who do you trust most for accurate information about the HPV vaccine?(doctor, teacher, religious leader, community leader or elder, family member, friend)	2 (item needs some revision)	2 (item needs some revision)	why not a nurse, a lady healthcare visitor, a midwife, a traditional birth attendant and so many more people involved in women's health
29. From which sources do you get information about the HPV vaccine? (religious leader, poster, healthcare worker, neighbour, social media, TV)	2 (item needs some revision)	2 (item needs some revision)	The same observation as item no 28

Motivation

	How relevant is this item	How clear is this item	Suggestions (if any)
30. When the HPV vaccine campaign begins, do you intend to register your daughter? (Extremely unlikely, Unlikely, Neutral, Likely, Extremely likely)	**4 (very relevant)**	**2 (item needs some revision)**	**why only daughter, why not myself or other women in my family?**
31.Do you believe vaccinating girls against HPV is as important as other childhood vaccines? (Strongly disagree, Disagree, Neutral, Agree, Strongly agree)	**4 (very relevant)**	**4 (very clear)**	
32. If you know that cervical cancer cases are increasing among young women in your area, will it motivate you to get your daughter vaccinated? (Strongly disagree, Disagree, Neutral, Agree, Strongly agree)	**2 (item needs some revision)**	**2 (item needs some revision)**	**same observation. women in your family**
33. If the government makes the HPV vaccine mandatory, will you get your daughter vaccinated? (Certainly no, Probably no, Neutral, Probably yes, Certainly yes)	**4 (very relevant)**	**2 (item needs some revision)**	**being free does not mean being accessible**
34. If any of your children has ever experienced side effects from routine vaccination, would you still decide to get your daughter vaccinated with the HPV vaccine? (Extremely unlikely, Unlikely, Neutral, Likely, Extremely likely)	**2 (item needs some revision)**	**2 (item needs some revision)**	**Item needs to be rephrased**
35. Compared to other vaccines, how much do you trust the safety of the HPV vaccine? (Not at all, To some extent, Neutral, To a considerable extent, To a great extent)	**4 (very relevant)**	**4 (very clear)**	
36. If you are given scientifically based information about the HPV vaccine, would you consider getting your daughter vaccinated? (Extremely unlikely, Unlikely, Neutral, Likely, Extremely likely)	**2 (item needs some revision)**	**2 (item needs some revision)**	**grammar needs correction**
37. If you learn that other countries are administering the HPV vaccine, would it increase your trust in vaccination? (Extremely unlikely, Unlikely, Neutral, Likely, Extremely likely)	**4 (very relevant)**	**4 (very clear)**	

Practical Issues

	How relevant is this item	How clear is this item	Suggestions (if any)
38. Would you get your daughter vaccinated even though the HPV vaccine is currently used on a limited scale in Pakistan?(Strongly disagree, Disagree, Neutral, Agree, Strongly agree)	2 (item needs some revision)	2 (item needs some revision)	item needs to be rephrased
39. If hospital staff provide limited information about the HPV vaccine, will you still choose to get your daughter vaccinated?(Extremely unlikely, Unlikely, Neutral, Likely, Extremely likely)	2 (item needs some revision)	2 (item needs some revision)	why only hospital staff, why not a primary healthcare center?
40. If government offers monetary benefits for getting the HPV vaccine, would people be more willing to get vaccinated?(Strongly disagree, Disagree, Neutral, Agree, Strongly agree)	2 (item needs some revision)	2 (item needs some revision)	Do you think monetization of vaccination is ethically appropriate?
41. Do you trust that the HPV vaccine will be safely administered in schools? (Not at all, To some extent, Neutral, To a considerable extent, To a great extent)	2 (item needs some revision)	2 (item needs some revision)	Question very unclear.
42. Do you think school administrations will actively support the HPV vaccination program? (Not at all, To some extent, Neutral, To a considerable extent, To a great extent)	2 (item needs some revision)	2 (item needs some revision)	Instead of school, the importance should be on legislation that makes it mandatory
43. Do you agree that public and private schools have different attitudes towards vaccination campaigns? (Not at all, To some extent, Neutral, To a considerable extent, To a great extent)	4 (very relevant)	4 (very clear)	
44. Do you think teachers should be given specific training to raise awareness about the HPV vaccine among adolescent girls?(Strongly disagree, Disagree, Neutral, Agree, Strongly agree)	2 (item needs some revision)	2 (item needs some revision)	are teachers given specific training for other vaccination programs?
45. Do you think girls should be given awareness about the HPV vaccine in schools? (Strongly disagree, Disagree, Neutral, Agree, Strongly agree)	2 (item needs some revision)	2 (item needs some revision)	Why only girls, why not boys. they need to be made aware as well.

	How relevant is this item	How clear is this item	Suggestions (if any)
46. Do you trust outreach vaccination services for your daughter's HPV vaccination? (Not at all, To some extent, Neutral, To a considerable extent, To a great extent)	2 (item needs some revision)	2 (item needs some revision)	replace daughetr with women in your family
47. Do you agree that people are concerned about the brand of the vaccine being used? (Not at all, To some extent, Neutral, To a considerable extent, To a great extent)	4 (very relevant)	3 (clear but needs minor revision)	locally manufactured versus imported.
48. Does the vaccination staff cooperate with you during the routine vaccination process? (Not at all, To some extent, Neutral, To a considerable extent, To a great extent)	2 (item needs some revision)	2 (item needs some revision)	Need to esatblish whether they have encountered any vaccination staff earleir. Maybe they don't have children and have never been to a vaccination center.
49. Where would you prefer your daughter to receive the HPV vaccine? (public hospital, private hospital, outreach service, school, others)	2 (item needs some revision)	2 (item needs some revision)	needs major revisions
50. What barriers do you think exist in your community regarding access to vaccines? (long distance, lack of information, affordability issues, work or family responsibilities, unavailability of the vaccine, lack of transport, clinic timings)	2 (item needs some revision)	2 (item needs some revision)	would recommend leaving it free text
51. How long do you usually wait at the health center for routine vaccination? (<10 min, 10 to 20 min, 21 to 30 min, >30 min)	4 (very relevant)	4 (very clear)	
52. Which social media platforms do you trust most for vaccine-related information? Select all that apply. (Tik-Tok, Facebook, Instagram, YouTube, Twitter, Other)	2 (item needs some revision)	2 (item needs some revision)	need to know whether they are using social media or not
53. How helpful do you think reminders (e.g. mobile messages, school announcements) are to ensure timely vaccination? (Extremely unlikely, Unlikely,	4 (very relevant)	4 (very clear)	

	How relevant is this item	How clear is this item	Suggestions (if any)
Neutral, Likely, Extremely likely)			
54. How often do you receive vaccine-related information through TV, radio or mobile phone? (Never, Rarely. Neutral, Sometimes, Always)	4 (very relevant)	4 (very clear)	

Cultural Integration

	How relevant is this item	How clear is this item	Suggestions (if any)
55. Who usually makes health decisions in your household? (Maternal grandparents, Paternal grandparents, Mother, Father, others)	4 (very relevant)	4 (very clear)	
56. Do you feel comfortable discussing reproductive health during family conversations? (Not at all, To some extent, Neutral, To a considerable extent, To a great extent)	2 (item needs some revision)	2 (item needs some revision)	question is culturally inappropriate
57. Do you think men should also be educated about the HPV vaccine through awareness campaigns?(Strongly disagree, Disagree, Neutral, Agree, Strongly agree)	4 (very relevant)	4 (very clear)	
58. Would you choose to get your daughter vaccinated against HPV even if your family opposes it? (Extremely unlikely, Unlikely, Neutral, Likely, Extremely likely)	2 (item needs some revision)	2 (item needs some revision)	question is culturally inappropriate
59. If your daughter wishes to receive the HPV vaccine but you have some reservations, how would you react? (Strongly oppose, Oppose, Neutral, Support, Fully support)	2 (item needs some revision)	2 (item needs some revision)	how can you measure a reaction, needs rephrasing
60. If people you know get their daughters vaccinated against HPV, would you be more willing to do the same? (Extremely unlikely, Unlikely, Neutral, Likely, Extremely likely)	4 (very relevant)	4 (very clear)	How will peer influence effect your decision to vaccinate
61. Do you think that hearing real stories of cervical cancer patients would increase parents' trust in the HPV vaccine? (Extremely unlikely,	2 (item needs some revision)	2 (item needs some revision)	why only parents, why not care givers or guardians?

	How relevant is this item	How clear is this item	Suggestions (if any)
Unlikely, Neutral, Likely, Extremely likely)			
62. If you see health care professionals vaccinating their own daughters against HPV, would you feel more confident about doing the same? (Extremely unlikely, Unlikely, Neutral, Likely, Extremely likely)	4 (very relevant)	4 (very clear)	
63. Do you agree that a female vaccinator should administer the HPV vaccine to girls? (Strongly disagree, Disagree, Neutral, Agree, Strongly agree)	2 (item needs some revision)	2 (item needs some revisio n)	The question should not put forth the investigators own opinions or thoughts/ agendas.
64. Do you agree that people in community hesitate to get the HPV vaccine due to social reasons? (Strongly disagree, Disagree, Neutral, Agree, Strongly agree)	2 (item needs some revision)	2 (item needs some revisio n)	Rephrase....Do people in community hesitate to get the HPV vaccine due to social reasons?
65. Do you think it is appropriate to choose only girls for the HPV vaccine? (Strongly disagree, Disagree, Neutral, Agree, Strongly agree)	2 (item needs some revision)	2 (item needs some revisio n)	very assumptive question
66. Do you think people from different ethnic backgrounds may hesitate to vaccinate their daughters against the HPV vaccine? (Not at all, To some extent, Neutral, To a considerable extent, To a great extent)	2 (item needs some revision)	2 (item needs some revisio n)	Pakistan has more than 200 ethnicities. which one are your referring to?
67. Would you agree that the HPV vaccine should be administered to your daughter at school without your permission? (Extremely unlikely, Unlikely, Neutral, Likely, Extremely likely)	2 (item needs some revision)	2 (item needs some revisio n)	Against medical ethics
