# Supplementary material for: Cultural adaptation and content validation of the WHO BeSD framework for HPV vaccination in Pakistan: A two-phase Delphi and cognitive interview study
Source: PLoS One. 2026 Feb 17;21(2):e0335358. doi: 10.1371/journal.pone.0335358 (PMC12912614; doi:10.1371/journal.pone.0335358)
Supplement: S4 File — 6309880716623591134. (PDF) [file pone.0335358.s004.pdf]

Thinking & Feeling

	How relevant is this item	How clear is this item	Suggestions (if any)
1. To what extent do you agree that early screening and preventive measures, such as vaccination, are necessary to protect women from serious illnesses?<br>(Not at all, To some extent, Neutral, To a considerable extent, To a great extent)	4 (very relevant)	4 (very)clear	
2. Do you agree that vaccines administered in Pakistan could be used for experimental purposes?<br>(Strongly disagree, Disagree, Neutral, Agree, Strongly agree)	3 (relevant but needs minor revision)	3 (clear but needs minor revision)	
3. **Have you ever heard that vaccines may contain haram (prohibited) ingredients such as pork?**<br>(Not at all, To some extent, Neutral, To a considerable extent, To a great extent)	4 (very relevant)	4 (very)clear	
4. Do you think the HPV vaccine is being introduced unnecessarily?(Not at all, To some extent, Neutral, To a considerable extent, To a great extent)	3 (relevant but needs minor revision)	3 (clear but needs minor revision)	
5. To what extent do you believe that the HPV vaccine protects against cervical cancer?<br>(Not at all, To some extent, Neutral, To a considerable extent, To a great extent)	4 (very relevant)	4 (very)clear	
6. Do you feel you are fully informed about the benefits of the HPV vaccine?<br>(Not at all, To some extent, Neutral, To a considerable extent, To a great extent)	4 (very relevant)	4 (very)clear	
7. How much do you trust the HPV vaccine being introduced in your country?(Not at all, To some extent, Neutral, To a considerable extent, To a great extent)	2 (item needs some revision)	2 (item needs some revision)	

	How relevant is this item	How clear is this item	Suggestions (if any)
8. Do you think that if the government makes the HPV vaccine mandatory, some people will try to get a vaccination card without actually being vaccinated? (Strongly disagree, Disagree, Neutral, Agree, Strongly agree)	**4 (very relevant)**	**4 (very)clear**	
9. If the HPV vaccine were offered free of cost, do you think people would hesitate to receive it? (Not at all hesitant, somewhat hesitant, Neutral, Quite hesitant, Extremely hesitant)	**4 (very relevant)**	**4 (very)clear**	
10. Do you think the HPV vaccine could be part of efforts to control the population? (Strongly disagree, Disagree, Neutral, Agree, Strongly agree)	**1 (not relevant)**	**1 (not clear)**	
11. Do you believe the HPV vaccine is acceptable in a Muslim society? (Strongly disagree, Disagree, Neutral, Agree, Strongly agree)	**4 (very relevant)**	**4 (very)clear**	
12. To what extent do you agree that all girls aged 9 to 14 years should be vaccinated against HPV?(Strongly disagree, Disagree, Neutral, Agree, Strongly agree)	**3 (relevant but needs minor revision)**	**3 (clear but needs minor revisio n)**	
13. If you believe your daughter is at low risk of cervical cancer, would you still get her vaccinated against HPV?(Certainly no, Probably no, Neutral, Probably yes, Certainly yes)	**4 (very relevant)**	**4 (very)clear**	
14. Do you think the HPV vaccine will help protect your daughter from serious illness? (Strongly disagree, Disagree, Neutral, Agree, Strongly agree)	**4 (very relevant)**	**4 (very)clear**	
15. **Do you think strong immunity reduces the importance of the HPV vaccine?**(Not at all, To some extent, Neutral, To a considerable extent, To a great extent)	**4 (very relevant)**	**4 (very)clear**	
16. **To what extent are you concerned about** the **possible** adverse effects of the HPV vaccine? (Not at all, To some extent, Neutral, To a considerable extent, To a great extent)	**4 (very relevant)**	**4 (very)clear**	
17. Do you think the HPV vaccine could negatively affect reproductive health?	**4 (very relevant)**	**4 (very)clear**	

	How relevant is this item	How clear is this item	Suggestions (if any)
(Strongly disagree, Disagree, Neutral, Agree, Strongly agree)			
18. How much do you trust social media to get information about the HPV vaccine? (Strongly disagree, Disagree, Neutral, Agree, Strongly agree)	**4 (very relevant)**	**4 (very)clear**	

Social Processes

	How relevant is this item	How clear is this item	Suggestions (if any)
19. How often do you hear rumors or misconceptions about the HPV vaccine in your community? (Never, Rarely. Neutral, Sometimes, Always)	**4 (very relevant)**	**4 (very clear)**	
20. To what extent do religious beliefs in your community influence decisions about the HPV vaccine? (Not at all, To some extent, Neutral, To a considerable extent, To a great extent)	**3 (relevant but needs minor revision)**	**3 (clear but needs minor revision)**	
21. Would people in your community trust information about the HPV vaccine available on social media? (Extremely unlikely, Unlikely, Neutral, Likely, Extremely likely)	**1 (not relevant)**	**1 (not clear)**	
22. Do you think married and unmarried women may have different opinions about the HPV vaccine? (Strongly disagree, Disagree, Neutral, Agree, Strongly agree)	**4 (very relevant)**	**4 (very clear)**	
23. Will you wait to observe its effects on others before deciding to get your daughter vaccinated? (Extremely unlikely, Unlikely, Neutral, Likely, Extremely likely)	**3 (relevant but needs minor revision)**	**3 (clear but needs minor revision)**	
24. If a trusted person in your community recommends the HPV vaccine, would you be more willing to get your daughter vaccinated? (Extremely unlikely, Unlikely, Neutral, Likely, Extremely likely)	**4 (very relevant)**	**4 (very clear)**	
25. Will you talk to your daughter about the HPV vaccine before deciding to get her vaccinated? (Extremely unlikely, Unlikely, Neutral, Likely, Extremely likely)	**4 (very relevant)**	**4 (very clear)**	
26. If you get your daughter vaccinated with the HPV vaccine,	**4 (very relevant)**	**4 (very clear)**	

	How relevant is this item	How clear is this item	Suggestions (if any)
would you recommend it to others as well?(Extremely unlikely, Unlikely, Neutral, Likely, Extremely likely)			
27. Do you prefer that the primary healthcare provider educating about the HPV vaccine be a female doctor?(Extremely unlikely, Unlikely, Neutral, Likely, Extremely likely)	**4 (very relevant)**	**4 (very clear)**	
28. Who do you trust most for accurate information about the HPV vaccine? (doctor, teacher, religious leader, community leader or elder, family member, friend)	**4 (very relevant)**	**4 (very clear)**	
29. From which sources do you get information about the HPV vaccine? (religious leader, poster, healthcare worker, neighbour, social media, TV)	**4 (very relevant)**	**4 (very clear)**	

Motivation

	How relevant is this item	How clear is this item	Suggestions (if any)
30. When the HPV vaccine campaign begins, do you intend to register your daughter?(Extremely unlikely, Unlikely, Neutral, Likely, Extremely likely)	**4 (very relevant)**	**4 (very clear)**	
31.Do you believe vaccinating girls against HPV is as important as other childhood vaccines? (Strongly disagree, Disagree, Neutral, Agree, Strongly agree)	**4 (very relevant)**	**4 (very clear)**	
32. If you know that cervical cancer cases are increasing among young women in your area, will it motivate you to get your daughter vaccinated? (Strongly disagree, Disagree, Neutral, Agree, Strongly agree)	**4 (very relevant)**	**4 (very clear)**	
33. If the government makes the HPV vaccine mandatory, will you get your daughter vaccinated? (Certainly no, Probably no, Neutral, Probably yes, Certainly yes)	**4 (very relevant)**	**4 (very clear)**	
34. If any of your children has ever experienced side effects from routine vaccination, would you still decide to get your daughter vaccinated with the HPV vaccine? (Extremely unlikely, Unlikely, Neutral, Likely, Extremely likely)	**3 (relevant but needs minor revision)**	**3 (clear but needs minor revisio n)**	
35. Compared to other vaccines, how much do you trust the safety of	**4 (very relevant)**	**4 (very clear)**	

	How relevant is this item	How clear is this item	Suggestions (if any)
the HPV vaccine?<br>(Not at all, To some extent, Neutral, To a considerable extent, To a great extent)			
36. If you are given scientifically based information about the HPV vaccine, would you consider getting your daughter vaccinated?<br>(Extremely unlikely, Unlikely, Neutral, Likely, Extremely likely)	**4 (very relevant)**	**4 (very clear)**	
37. If you learn that other countries are administering the HPV vaccine, would it increase your trust in vaccination?<br>(Extremely unlikely, Unlikely, Neutral, Likely, Extremely likely)	**4 (very relevant)**	**4 (very clear)**	

Practical Issues

	How relevant is this item	How clear is this item	Suggestions (if any)
38. Would you get your daughter vaccinated even though the HPV vaccine is currently used on a limited scale in Pakistan?(Strongly disagree, Disagree, Neutral, Agree, Strongly agree)	**4 (very relevant)**	**4 (very clear)**	
39. If hospital staff provide limited information about the HPV vaccine, will you still choose to get your daughter vaccinated?(Extremely unlikely, Unlikely, Neutral, Likely, Extremely likely)	**3 (relevant but needs minor revision)**	**3 (clear but needs minor revision)**	
40. If government offers monetary benefits for getting the HPV vaccine, would people be more willing to get vaccinated?(Strongly disagree, Disagree, Neutral, Agree, Strongly agree)	**4 (very relevant)**	**4 (very clear)**	
41. Do you trust that the HPV vaccine will be safely administered in schools?<br>(Not at all, To some extent, Neutral, To a considerable extent, To a great extent)	**4 (very relevant)**	**4 (very clear)**	
42. Do you think school administrations will actively support the HPV vaccination program?<br>(Not at all, To some extent, Neutral, To a considerable extent, To a great extent)	**4 (very relevant)**	**4 (very clear)**	
43. Do you agree that public and private schools have different	**4 (very relevant)**	**4 (very clear)**	

	How relevant is this item	How clear is this item	Suggestions (if any)
attitudes towards vaccination campaigns?<br>(Not at all, To some extent, Neutral, To a considerable extent, To a great extent)			
44. Do you think teachers should be given specific training to raise awareness about the HPV vaccine among adolescent girls?(Strongly disagree, Disagree, Neutral, Agree, Strongly agree)	**4 (very relevant)**	**4 (very clear)**	
45. Do you think girls should be given awareness about the HPV vaccine in schools?<br>(Strongly disagree, Disagree, Neutral, Agree, Strongly agree)	**4 (very relevant)**	**4 (very clear)**	
46. Do you trust outreach vaccination services for your daughter's HPV vaccination?<br>(Not at all, To some extent, Neutral, To a considerable extent, To a great extent)	**4 (very relevant)**	**4 (very clear)**	
47. Do you agree that people are concerned about the brand of the vaccine being used?<br>(Not at all, To some extent, Neutral, To a considerable extent, To a great extent)	**3 (relevant but needs minor revision)**	**3 (clear but needs minor revision)**	
48. Does the vaccination staff cooperate with you during the routine vaccination process?<br>(Not at all, To some extent, Neutral, To a considerable extent, To a great extent)	**4 (very relevant)**	**4 (very clear)**	
49. Where would you prefer your daughter to receive the HPV vaccine?<br>(public hospital, private hospital, outreach service, school, others)	**4 (very relevant)**	**4 (very clear)**	
50. What barriers do you think exist in your community regarding access to vaccines? (long distance, lack of information, affordability issues, work or family responsibilities, unavailability of the vaccine, lack of transport, clinic timings)	**4 (very relevant)**	**4 (very clear)**	
51. How long do you usually wait at the health center for routine vaccination? (<10 min, 10 to 20 min, 21 to 30 min, >30 min)	**3 (relevant but needs minor revision)**	**3 (clear but needs minor revision)**	
52. Which social media platforms do you trust most for vaccine-related	**4 (very relevant)**	**4 (very clear)**	

	How relevant is this item	How clear is this item	Suggestions (if any)
information? Select all that apply. (Tik-Tok, Facebook, Instagram, YouTube, Twitter, Other)			
53. How helpful do you think reminders (e.g. mobile messages, school announcements) are to ensure timely vaccination? (Extremely unlikely, Unlikely, Neutral, Likely, Extremely likely)	**4 (very relevant)**	**4 (very clear)**	
54. How often do you receive vaccine-related information through TV, radio or mobile phone? (Never, Rarely. Neutral, Sometimes, Always)	**4 (very relevant)**	**4 (very clear)**	

Cultural Integration

	How relevant is this item	How clear is this item	Suggestions (if any)
55. Who usually makes health decisions in your household? (Maternal grandparents, Paternal grandparents, Mother, Father, others)	**3 (relevant but needs minor revision)**	**3 (clear but needs minor revision)**	
56. Do you feel comfortable discussing reproductive health during family conversations? (Not at all, To some extent, Neutral, To a considerable extent, To a great extent)	**3 (relevant but needs minor revision)**	**3 (clear but needs minor revision)**	
57. Do you think men should also be educated about the HPV vaccine through awareness campaigns? (Strongly disagree, Disagree, Neutral, Agree, Strongly agree)	**4 (very relevant)**	**4 (very clear)**	
58. Would you choose to get your daughter vaccinated against HPV even if your family opposes it? (Extremely unlikely, Unlikely, Neutral, Likely, Extremely likely)	**4 (very relevant)**	**4 (very clear)**	
59. If your daughter wishes to receive the HPV vaccine but you have some reservations, how would you react? (Strongly oppose, Oppose, Neutral, Support, Fully support)	**2 (item needs some revision)**	**2 (item needs some revision)**	
60. If people you know get their daughters vaccinated against HPV, would you be more willing to do the same? (Extremely unlikely, Unlikely, Neutral, Likely, Extremely likely)	**4 (very relevant)**	**4 (very clear)**	
61. Do you think that hearing real stories of cervical cancer patients	**4 (very relevant)**	**4 (very clear)**	

	How relevant is this item	How clear is this item	Suggestions (if any)
would increase parents' trust in the HPV vaccine? (Extremely unlikely, Unlikely, Neutral, Likely, Extremely likely)			
62. If you see health care professionals vaccinating their own daughters against HPV, would you feel more confident about doing the same?(Extremely unlikely, Unlikely, Neutral, Likely, Extremely likely)	**4 (very relevant)**	**4 (very clear)**	
63. Do you agree that a female vaccinator should administer the HPV vaccine to girls?(Strongly disagree, Disagree, Neutral, Agree, Strongly agree)	**4 (very relevant)**	**4 (very clear)**	
64. Do you agree that people in community hesitate to get the HPV vaccine due to social reasons? (Strongly disagree, Disagree, Neutral, Agree, Strongly agree)	**4 (very relevant)**	**4 (very clear)**	
65. Do you think it is appropriate to choose only girls for the HPV vaccine? (Strongly disagree, Disagree, Neutral, Agree, Strongly agree)	**4 (very relevant)**	**4 (very clear)**	
66. Do you think people from different ethnic backgrounds may hesitate to vaccinate their daughters against the HPV vaccine? (Not at all, To some extent, Neutral, To a considerable extent, To a great extent)	**4 (very relevant)**	**4 (very clear)**	
67. Would you agree that the HPV vaccine should be administered to your daughter at school without your permission? (Extremely unlikely, Unlikely, Neutral, Likely, Extremely likely)	**4 (very relevant)**	**4 (very clear)**	
